# Supplementary material for: Blue and green food webs respond differently to elevation and land use
Source: Nat Commun. 2022 Oct 27;13:6415. doi: 10.1038/s41467-022-34132-9 (PMC9613893; doi:10.1038/s41467-022-34132-9)
Supplement: Supplementary file 1 — Supplementary Information [file 41467_2022_34132_MOESM1_ESM.pdf]

SUPPLEMENTARY INFORMATION

Blue and green food webs respond differently to elevation and land use

Hsi-Cheng Ho[1,*], Jakob Brodersen[2], Martin M. Gossner[3, 4], Catherine H. Graham[3], Silvana Kaeser[1], Merin Reji Chacko[3, 4], Ole Seehausen[2, 5], Niklaus E. Zimmermann[3, 4], Loïc Pellissier[3, 4], and Florian Altermatt[1, 6,*]

[1] Department of Aquatic Ecology, Eawag: Swiss Federal Institute of Aquatic Science and Technology, Überlandstrasse 133, CH-8600 Dübendorf, Switzerland

[2] Department Fish Ecology and Evolution, Eawag: Swiss Federal Institute of Aquatic Science and Technology, Seestrasse 79, CH-6047 Kastanienbaum, Switzerland

[3] WSL Swiss Federal Research Institute, Zürcherstrasse 111, CH-8903 Birmensdorf, Switzerland

[4] Department of Environmental Systems Science, Institute of Terrestrial Ecosystems, ETH Zürich, Universitätstrasse 16, CH-8092 Zürich, Switzerland

[5] Division Aquatic Ecology, Institute of Ecology and Evolution, University of Bern, Baltzerstrasse 6, CH-3012 Bern, Switzerland

[6] Department of Evolutionary Biology and Environmental Studies, University of Zürich, Winterthurerstrasse 190, CH-8057 Zürich, Switzerland

*** Corresponding authors:**

Hsi-Cheng Ho (hsichengho@gmail.com)

Florian Altermatt (Florian.Altermatt@eawag.ch)

Supplementary Methods

The occurrence data and the metaweb

Regarding the occurrence data we used, here we provide a summarising table (Supplementary Table 1) of the sources, taxonomic resolutions, and time frames of the collected datasets. All datasets' occurrence information well-covered the area of Switzerland (*BDM* & *info fauna* on a grid basis; *Progetto Fiumi* on a site basis across Swiss streams, and we converted it to grids as in the formers). To access the data, or for more detailed information regarding the occurrence survey, please contact corresponding authorities: Biodiversitätsmonitoring Schweiz (*BDM*), Centre Suisse de Cartographie de la Faune, CSCF (*info fauna*), and Jakob Brodersen (*Progetto Fiumi*), respectively.

Supplementary Table 1. Summarising table of the occurrence data.

**Taxa group**	**Data source**	**Taxonomic resolution**	**Time frame**
Plant	*BDM*	Species/Species-complex	2001–2013; 2016–2020
Butterfly	*BDM*	Species/Species-complex	2001–2013; 2016–2020
Grasshopper	*info fauna*	Species	1990–2020
Bird	*BDM*	Species	2001–2013; 2016–2020
Aquatic Inv.	*BDM*	Species/Family	2009–2014
Fish	*info fauna*	Species (some aggregations)	1990–2020
Fish	*Progetto Fiumi*	Species	2013–2017

Regarding the trophic interaction information, we here provide a meta-data table (Supplementary Table 2) of our metaweb. The majority of trophic interactions among focal groups were first assigned based on the listed references (which themselves are based on natural history observations), then further complemented or refined by knowledge from the authors' and collaborators' empirical research experiences or unpublished data. The metaweb *per se* as tables of consumer-resource links can be accessed at the provided online repository, and the sources of information are noted there. Note that cannibalistic links were not included in this study.

Supplementary Table 2: Meta-data of the metaweb.

**Trophic information**	**Taxonomic scale**	**References** (ordered by year)
Butterfly-plant (larvae-host) interactions	Species/Sp.-complex	Ebert (1991–2005)<br>Landolt et al. (2010)
Grasshoppers' diets	Species	Detzel (1998)<br>Ingrisch & Köhler (1998)<br>Maas et al. (2002)<br>Schlumprecht & Waeber (2003)<br>Baur et al. (2006)<br>Landolt et al. (2010)<br>Klaiber et al. (2017)<br>Pitteloud et al. (2020)
Birds' diets	Species	Storchová & Hořák (2018)<br>Vogelwarte.ch
Aquatic invertebrates feeding group assignment and trophic relationships among groups	Species/Family	Moog (1995)<br>Schmedtje & Colling (1996)<br>*AQEM expert consortium* (2002)<br>Graf et al. (2008; 2009)<br>Tachet et al. (2010)<br>*freshwaterecology.info*: Schmidt-Kloiber & Hering (2015)
Fishes' diets	Species/Sp.-complex	Kottelat & Freyhof (2007)<br>*FishBase*: Froese & Pauly (2010)

Food-web metrics quantifying and analysing tools

All metric quantification and analyses were done in R language. For quantifying food-web nestedness, we used the *unodf* function from *UNODF* package (Cantor et al. 2017). For quantifying food-web modularity, we used in combination the *graph.adjacency*, *multilevel.community*, and *modularity* functions from *igraph* package (Csardi & Nepusz 2006). For quantifying diet niche overlap of the consumers, we adopted the inbuilt Horn's index calculation of the *networklevel* function from *bipartite* package (Dormann et al. 2009).

We carried out the principal component analysis using R-base *prcomp* function, then visualised the result using the *ggbiplot* function from *ggbiplot* package (Vu 2011). The general linear model analyses were conducted using R-base *lm* and *anova* functions, while the structural equation modelling analyses using the *psem* function from *piecewiseSEM* package (Lefcheck 2016). The linear mixed models embedded in the structural equation modelling analyses were applying the *lmer* function of *lme4* package (Bates et al. 2015). The linear-model assumptions of the structural equation modelling were tested using the *xyplot* and *qqmath* functions of *lattice* package (Sarkar 2008) for linear mixed model cases, whereas the R base *plot* function for linear model cases. We plotted food-web metrics against elevation, and performed the relevant generalised additive models and linear models analyses, using the ggplot2 package (Wickham 2016) and its inbuilt analysing functions. The land-use-specific linear model slope comparisons were carried out using the *lm* function and *lstrends* function of *lsmeans* package (Lenth 2016) in combination. The assumptions for these linear models were tested using the *gam* function of *mgcv* package (Wood 2011).

We illustrated the location and composition of our food webs on a Swiss map (area frame) using the *geom_scatterpie* function from *scatterpie* package (Yu 2021) and function *png* from *png* package (Urbanek 2013). The *ggpubr* (Kassabara 2020), *ggpmisc* (Aphalo 2020), and *gridExtra* (Auguie 2017) packages were also applied alongside *ggplot2* for generating needed components of the plots.

Supplementary Tables

Supplementary Table 3. General linear model analyses with observed properties of the inferred food webs as response variable, while elevation ("Ele", continuous), system ("Sys", 2 levels: terrestrial versus aquatic), and dominant land-use type ("DLT", 5 levels: forests, scrubs, open spaces, farmlands, urban) as interacting predictors. Two-tailed significance code: * P < 0.05, ** P < 0.01, *** P < 0.001.

Model: Response variable ~ Elevation (Ele) * System (Sys) * Dominant Land-use Type (DLT)

**No.Nodes**	Df	F value	P value
Ele	1	142.987	< 2.2e-16 ***
Sys	1	13687.882	< 2.2e-16 ***
DLT	4	91.803	< 2.2e-16 ***
Ele * Sys	1	151.173	< 2.2e-16 ***
Ele * DLT	4	29.586	< 2.2e-16 ***
Sys * DLT	4	49.350	< 2.2e-16 ***
Ele * Sys * DLT	4	21.416	< 2.2e-16 ***

**Connectance**	Df	F value	P value
Ele	1	332.531	< 2.2e-16 ***
Sys	1	10798.702	< 2.2e-16 ***
DLT	4	11.302	6.2e-9 ***
Ele * Sys	1	0.787	0.375
Ele * DLT	4	9.253	2.6e-7 ***
Sys * DLT	4	10.468	2.8e-8 ***
Ele * Sys * DLT	4	15.259	4.8e-12 ***

**Nestedness**	Df	F value	P value
Ele	1	232.542	< 2.2e-16 ***
Sys	1	3.965	0.047 *
DLT	4	11.558	3.9e-9 ***
Ele * Sys	1	59.518	3.5e-14 ***
Ele * DLT	4	4.097	0.003 **
Sys * DLT	4	10.191	4.7e-8 ***
Ele * Sys * DLT	4	11.517	4.2e-9 ***

**Modularity**	Df	F value	P value
Ele	1	417.484	< 2.2e-16 ***
Sys	1	8849.818	< 2.2e-16 ***
DLT	4	7.814	3.5e-6 ***
Ele * Sys	1	127.811	< 2.2e-16 ***
Ele * DLT	4	2.432	0.046 *
Sys * DLT	4	10.401	3.2e-8 ***
Ele * Sys * DLT	4	2.791	0.025 *

**Niche Overlap**	Df	F value	P value
Ele	1	159.672	< 2.2e-16 ***
Sys	1	12488.197	< 2.2e-16 ***
DLT	4	3.470	0.008 **
Ele * Sys	1	163.613	< 2.2e-16 ***
Ele * DLT	4	3.586	0.007 **
Sys * DLT	4	27.501	< 2.2e-16 ***
Ele * Sys * DLT	4	20.263	6.1e-16 ***

Supplementary Table 4. General linear model analyses with observed properties of the inferred food webs as response variable, while elevation ("Ele", continuous) and the residual temperature ("ResTem", continuous) as independent predictors. The residual temperature is derived from removing the linear regression main effects of elevation on temperature. We note that elevation is the better predictor over residual temperature throughout. Two-tailed significance code: ** P < 0.01, *** P < 0.001.

Model: Response variable ~ Elevation (Ele) + Residual Temperature (ResTem)

**No.Nodes**	Df	F value	P value
Ele	1	7.855	0.005 **
ResTem	1	0.099	0.753

**Connectance**	Df	F value	P value
Ele	1	23.869	1.2e-6 ***
ResTem	1	0.125	0.724

**Nestedness**	Df	F value	P value
Ele	1	189.363	< 2.2e-16 ***
ResTem	1	2.094	0.148

**Modularity**	Df	F value	P value
Ele	1	35.767	3.3e-9 ***
ResTem	1	0.364	0.546

**Niche Overlap**	Df	F value	P value
Ele	1	9.882	0.002 **
ResTem	1	0.002	0.961

Supplementary Table 5. Linearity tests of land-use type specific food-web metrics against elevation in the terrestrial system. The tests fit the data to generalised additive models (GAM) with a smooth term having a penalty on its 2nd derivative and no null space (Wood 2006). Significant smooth terms (P value) indicate nonlinear relationships. An 1—2 estimated degrees of freedom (edf) indicates a weak nonlinearity, while >2 a strong one (Zuur et al. 2009). Nonlinearity was detected in individual cases but mostly weak. The rest majority was linear. Thus, based on the principle of parsimony, we analysed the elevational patterns of these (land-use subsetted) metrics with linear models (as stated in main text *Methods* and corresponding to Supplementary Figures 3 & 4). Same for the aquatic system (the next table). Two-tailed significance code: ** P < 0.01, *** P < 0.001.

Model: Response variable ~ Elevation (Ele) + Smooth term with no null space

**No.Nodes**				
**Land-use**	**Smooth term stats**			
	edf	R.df	F value	P value
Forests	1.717	8	1.425	< 0.001 ***
Scrubs	1.854	8	1.232	0.003 **
Open spaces	< 0.001	8	< 0.001	0.489
Farmlands	1.043	8	0.301	0.093
Urban	< 0.001	8	< 0.001	0.634

**Connectance**				
**Land-use**	**Smooth term stats**			
	edf	R.df	F value	P value
Forests	1.708	8	1.202	0.002 **
Scrubs	< 0.001	8	< 0.001	0.717
Open spaces	0.939	8	0.311	0.077
Farmlands	1.064	8	0.372	0.058
Urban	0.972	8	0.351	0.085

**Nestedness**				
**Land-use**	**Smooth term stats**			
	edf	R.df	F value	P value
Forests	< 0.001	8	< 0.001	0.577
Scrubs	1.289	8	0.333	0.115
Open spaces	< 0.001	8	< 0.001	0.425
Farmlands	0.001	8	< 0.001	0.375
Urban	< 0.001	8	< 0.001	0.733

**Modularity**				
**Land-use**	**Smooth term stats**			
	edf	R.df	F value	P value
Forests	3.955	8	2.045	0.001 **
Scrubs	< 0.001	8	< 0.001	0.904
Open spaces	1.017	8	0.347	0.070
Farmlands	1.476	8	0.336	0.143
Urban	< 0.001	8	< 0.001	0.536

**Niche Overlap**				
**Land-use**	**Smooth term stats**			
	edf	R.df	F value	P value
Forests	2.902	8	3.605	< 0.001 ***
Scrubs	3.366	8	2.028	0.001 **
Open spaces	< 0.001	8	< 0.001	0.497
Farmlands	0.025	8	0.003	0.325
Urban	< 0.001	8	< 0.001	0.567

Supplementary Table 6. Linearity tests of land-use type specific food-web metrics against elevation in the aquatic system. Please see the caption of Supplementary Table 5 for more details. Two-tailed significance code: * P < 0.05, ** P < 0.01, *** P < 0.001.

Model: Response variable ~ Elevation (Ele) + Smooth term with no null space

No.Nodes

**Land-use**	**Smooth term stats**			
	edf	R.df	F value	P value
Forests	< 0.001	8	< 0.001	0.748
Scrubs	1.651	8	0.612	0.041 *
Open spaces	< 0.001	8	< 0.001	0.778
Farmlands	1.372	8	0.682	0.017 *
Urban	1.198	8	0.578	0.030 *

Connectance

**Land-use**	**Smooth term stats**			
	edf	R.df	F value	P value
Forests	3.316	8	4.007	< 0.001 ***
Scrubs	< 0.001	8	< 0.001	0.837
Open spaces	0.425	8	0.071	0.256
Farmlands	2.091	8	2.232	< 0.001 ***
Urban	0.894	8	0.279	0.096

Nestedness

**Land-use**	**Smooth term stats**			
	edf	R.df	F value	P value
Forests	3.317	8	3.004	< 0.001 ***
Scrubs	0.001	8	< 0.001	0.373
Open spaces	< 0.001	8	< 0.001	0.505
Farmlands	1.251	8	0.629	0.018 *
Urban	0.998	8	0.337	0.080

Modularity

**Land-use**	**Smooth term stats**			
	edf	R.df	F value	P value
Forests	0.909	8	0.255	0.102
Scrubs	1.477	8	0.789	0.012 *
Open spaces	< 0.001	8	< 0.001	0.677
Farmlands	2.146	8	1.202	0.005 **
Urban	< 0.001	8	< 0.001	0.632

Niche Overlap

**Land-use**	**Smooth term stats**			
	edf	R.df	F value	P value
Forests	2.174	8	2.272	< 0.001 ***
Scrubs	< 0.001	8	< 0.001	0.616
Open spaces	< 0.001	8	< 0.001	0.940
Farmlands	2.402	8	2.941	< 0.001 ***
Urban	0.882	8	0.282	0.091

Supplementary Table 7. Stats of piecewise SEM on food webs whose dominant land-use type can be defined (n = 421 in terrestrial and n = 430 in aquatic system). The SEM dependency structure and the reported standardised estimates are corresponding to main text Figs. 3b–3c. Two-tailed significance code: · P < 0.1, * P < 0.05, ** P < 0.01, *** P < 0.001.

Overall model (set dominant land-use type as a random effect)										
	Terrestrial					Aquatic				
**SEM dependencies**	Est./ Std.Est.	Std. Error	Df	t value	P value	Est./ Std.Est.	Std. Error	Df	t value	P value
Elevation → No.Nodes	-0.054/ -0.335	0.010	363.8	-5.332	< 0.001 ***	-0.010/ -0.561	7.2e-4	8.2	-14.02	< 0.001 ***
Elevation → Connectance	-1.1e-5/ -0.539	1.3e-6	341.5	-8.712	< 0.001 ***	-1.6e-5/ -0.253	5.4e-6	93.5	-3.035	0.006 **
No.Nodes → Connectance	-4.3e-5/ -0.331	6.1e-6	417.2	-7.065	< 0.001 ***	2.6e-5/ 0.007	2.0e-4	423.4	0.132	0.895
Elevation → Nestedness	3.3e-6/ 0.075	3.0e-6	81.4	1.120	0.292	-1.4e-5/ -0.099	4.1e-6	28.8	-3.304	0.005 **
No.Nodes → Nestedness	10e-5/ 0.363	1.4e-5	42.5	7.226	< 0.001 ***	3.1e-3/ 0.399	1.8e-4	423.3	17.47	< 0.001 ***
Connectance → Nestedness	0.952/ 0.447	0.120	268.3	7.911	< 0.001 ***	1.523/ 0.709	0.043	405.9	35.48	< 0.001 ***
Elevation → Modularity	3.1e-5/ 0.490	4.9e-6	220.6	6.216	< 0.001 ***	-8.1e-6/ -0.379	1.4e-6	36.2	-5.864	< 0.001 ***
No.Nodes → Modularity	7.9e-5/ 0.204	2.3e-5	314.5	3.459	< 0.001 ***	-8.1e-4/ -0.672	5.7e-5	423.0	-14.28	< 0.001 ***
Connectance → Modularity	-0.119/ -0.040	0.176	400.5	-0.673	0.507	-0.045/ -0.135	0.014	417.3	-3.276	0.001 **
Elevation → Niche Overlap	-3.4e-5/ -0.533	3.0e-6	398.8	-11.04	< 0.001 ***	9.1e-6/ 0.102	4.6e-6	39.6	1.971	0.079 ·
No.Nodes → Niche Overlap	-1.7e-4/ -0.443	1.4e-5	410.3	-12.08	< 0.001 ***	-1.3e-3/ -0.267	2.8e-4	421.5	-4.689	< 0.001 ***
Connectance → Niche Overlap	1.525/ 0.507	0.109	415.0	14.05	< 0.001 ***	-1.626/ -1.173	0.090	423.3	-18.13	< 0.001 ***
Nestedness → Niche Overlap	0.139/ 0.098	0.039	411.3	3.573	< 0.001 ***	0.397/ 0.616	0.050	423.0	7.893	< 0.001 ***
Modularity → Niche Overlap	-0.057/ -0.056	0.028	413.1	-2.018	0.044 *	-0.480/ -0.115	0.157	423.9	-3.050	0.003 **
Global goodness-of-fit	Fisher's C = 2.988, P = 0.224 on 2 Df					Fisher's C = 20.196, P < 0.001 on 2 Df (see below †)				

† The analysis' test of directed separation suggested a direct effect from nestedness to modularity (i.e., corresponding to the SEM structure as shown in Supplementary Figure 2). However, adding such a direct path would eliminate all independence claims (global Fisher's C = 0 without degrees of freedom). As stated in Supplementary Figure 2 caption, given the qualitatively and quantitatively consistent path values, we reported the aquatic overall SEM model as shown here, sticking with our pre-defined SEM structure.

Supplementary Table 8. Stats of piecewise SEM on food webs whose dominant land-use type is forest (n = 152 in terrestrial and n = 135 in aquatic system). The SEM dependency structure and the reported standardised estimates are corresponding to Supplementary Figure 1. Two-tailed significance code: · P < 0.1, * P < 0.05, ** P < 0.01, *** P < 0.001.

Land-use type specific model (analyse subset food webs with dominant land use as forest)										
	Terrestrial					Aquatic				
**SEM dependencies**	Est./ Std.Est.	Std. Error	Df	t value	P value	Est./ Std.Est.	Std. Error	Df	t value	P value
Elevation → No.Nodes	-0.039/ -0.229	0.014	150	-2.879	0.005 **	-0.012/ -0.474	0.002	133	-6.20	< 0.001 ***
Elevation → Connectance	-8.4e-6/ -0.389	1.5e-6	149	-5.800	< 0.001 ***	-8.2e-6/ -0.099	8.1e-6	132	-1.013	0.313
No.Nodes → Connectance	-7.1e-5/ -0.558	8.5e-6	149	-8.307	< 0.001 ***	2.5e-4/ 0.077	3.2e-4	132	0.787	0.433
Elevation → Nestedness	-6.6e-6/ -0.116	5.0e-6	148	-1.316	0.190	-5.3e-6/ -0.030	7.3e-6	131	-0.723	0.471
No.Nodes → Nestedness	1.0e-4/ 0.309	3.2e-5	148	3.218	0.002 **	3.5e-3/ 0.491	2.9e-4	131	11.98	< 0.001 ***
Connectance → Nestedness	0.431/ 0.163	0.257	148	1.681	0.095 ·	1.494/ 0.696	0.078	131	19.12	< 0.001 ***
Elevation → Modularity	4.0e-5/ 0.465	7.3e-6	148	5.472	< 0.001 ***	-3.4e-6/ -0.145	1.9e-6	131	-1.738	0.085 ·
No.Nodes → Modularity	1.4e-4/ 0.286	4.7e-5	148	3.081	0.003 **	-5.4e-4/ -0.583	7.7e-5	131	-7.018	< 0.001 ***
Connectance → Modularity	0.855/ 0.214	0.374	148	2.288	0.024 *	-0.027/ -0.098	0.021	131	-1.329	0.186
Elevation → Niche Overlap	-3.2e-5/ -0.375	3.8e-6	146	-8.278	< 0.001 ***	2.0e-6/ 0.018	5.5e-6	129	0.364	0.716
No.Nodes → Niche Overlap	-8.9e-5/ -0.180	2.4e-5	146	-3.715	< 0.001 ***	-1.8e-3/ -0.424	3.4e-4	129	-5.393	< 0.001 ***
Connectance → Niche Overlap	2.378/ 0.609	0.183	146	13.02	< 0.001 ***	-1.722/ -1.312	0.113	129	-15.28	< 0.001 ***
Nestedness → Niche Overlap	0.087/ 0.059	0.057	146	1.526	0.129	0.426/ 0.697	0.064	129	6.613	< 0.001 ***
Modularity → Niche Overlap	-0.125/ -0.128	0.039	146	-3.191	0.002 **	-0.775/ -0.165	0.244	129	-3.181	0.002 **
Global goodness-of-fit	Fisher's C = 3.773, P = 0.152 on 2 Df					Fisher's C = 0.953, P = 0.621 on 2 Df				

Supplementary Table 9. Stats of piecewise SEM on food webs whose dominant land-use type is scrub (n = 63 in terrestrial and n = 78 in aquatic system). The SEM dependency structure and the reported standardised estimates are corresponding to Supplementary Figure 1. Two-tailed significance code: · P < 0.1, * P < 0.05, ** P < 0.01, *** P < 0.001.

Land-use type specific model (analyse subset food webs with dominant land use as scrub)										
	Terrestrial					Aquatic				
**SEM dependencies**	Est./ Std.Est.	Std. Error	Df	t value	P value	Est./ Std.Est.	Std. Error	Df	t value	P value
Elevation → No.Nodes	-0.181/ -0.674	0.025	61	-7.133	< 0.001 ***	-0.009/ -0.313	0.003	76	-2.870	0.005 **
Elevation → Connectance	-2.6e-5/ -0.825	4.2e-6	60	-6.080	< 0.001 ***	4.1e-5/ 0.431	1.1e-5	75	3.895	< 0.001 ***
No.Nodes → Connectance	-4.5e-5/ -0.390	1.6e-5	60	-2.878	0.006 **	2.7e-4/ 0.080	3.7e-4	75	0.726	0.470
Elevation → Nestedness	-1.0e-6/ -0.016	1.2e-5	59	-0.085	0.933	-2.3e-6/ -0.012	9.4e-6	74	-0.245	0.807
No.Nodes → Nestedness	2.5e-5/ 0.106	3.8e-5	59	0.663	0.510	3.4e-3/ 0.516	3.1e-4	74	11.15	< 0.001 ***
Connectance → Nestedness	0.996/ 0.487	0.292	59	3.416	0.001 **	1.564/ 0.801	0.094	74	16.67	< 0.001 ***
Elevation → Modularity	2.1e-5/ 0.253	1.8e-5	59	1.151	0.254	-7.1e-6/ -0.140	4.1e-6	74	-1.744	0.085 ·
No.Nodes → Modularity	8.0e-6/ 0.026	5.7e-5	59	0.140	0.889	-1.4e-3/ -0.785	1.3e-4	74	-10.66	< 0.001 ***
Connectance → Modularity	0.245/ 0.092	0.438	59	0.559	0.578	-0.138/ -0.259	0.041	74	-3.392	0.001 **
Elevation → Niche Overlap	3.1e-6/ 0.048	8.9e-6	57	0.351	0.727	2.1e-5/ 0.133	1.0e-5	72	2.074	0.042 *
No.Nodes → Niche Overlap	-7.7e-5/ -0.317	2.8e-5	57	-2.776	0.007 **	-2.7e-3/ -0.492	6.4e-4	72	-4.261	< 0.001 ***
Connectance → Niche Overlap	1.436/ 0.690	0.231	57	6.204	< 0.001 ***	-2.285/ -1.386	0.217	72	-10.54	< 0.001 ***
Nestedness → Niche Overlap	0.195/ 0.192	0.097	57	2.010	0.049 *	0.441/ 0.522	0.123	72	3.596	< 0.001 ***
Modularity → Niche Overlap	0.061/ 0.079	0.065	57	0.950	0.346	-0.806/ -0.260	0.283	72	-2.850	0.006 **
Global goodness-of-fit	Fisher's C = 5.141, P = 0.077 on 2 Df					Fisher's C = 1.157, P = 0.561 on 2 Df				

Supplementary Table 10. Stats of piecewise SEM on food webs whose dominant land-use type is open space (n = 52 in terrestrial and n = 26 in aquatic system). The SEM dependency structure and the reported standardised estimates are corresponding to Supplementary Figure 1. Two-tailed significance code: · P < 0.1, * P < 0.05, ** P < 0.01, *** P < 0.001.

Land-use type specific model (analyse subset food webs with dominant land use as open space)										
	Terrestrial					Aquatic				
**SEM dependencies**	Est./ Std.Est.	Std. Error	Df	t value	P value	Est./ Std.Est.	Std. Error	Df	t value	P value
Elevation → No.Nodes	-0.263/ -0.667	0.042	50	-6.326	< 0.001 ***	-0.016/ -0.570	4.6e-3	24	-3.402	0.002 **
Elevation → Connectance	-1.7e-5/ -0.256	1.2e-5	49	-1.362	0.180	1.1e-5/ 0.128	2.2e-5	23	0.508	0.616
No.Nodes → Connectance	-2.3e-5/ -0.137	3.1e-5	49	-0.728	0.470	2.6e-5/ 0.008	8.1e-4	23	0.033	0.974
Elevation → Nestedness	3.9e-5/ 0.200	3.0e-5	48	1.322	0.193	3.9e-5/ 0.157	3.1e-5	22	1.251	0.224
No.Nodes → Nestedness	2.3e-4/ 0.462	7.4e-5	48	3.097	0.003 **	6.1e-3/ 0.678	1.1e-3	22	5.428	< 0.001 ***
Connectance → Nestedness	1.630/ 0.548	0.336	48	4.860	< 0.001 ***	1.860/ 0.662	0.291	22	6.404	< 0.001 ***
Elevation → Modularity	-6.3e-5/ -0.346	3.3e-5	48	-1.940	0.058 ·	-1.8e-6/ -0.043	1.0e-5	22	-0.168	0.868
No.Nodes → Modularity	-1.2e-4/ -0.257	8.2e-5	48	-1.461	0.151	-3.1e-4/ -0.209	3.8e-4	22	-0.826	0.418
Connectance → Modularity	-1.096/ -0.394	0.370	48	-2.963	0.005 **	0.050/ 0.106	0.099	22	0.504	0.619
Elevation → Niche Overlap	1.4e-5/ 0.074	1.8e-5	46	0.805	0.425	-3.4e-5/ -0.108	3.6e-5	20	-0.941	0.358
No.Nodes → Niche Overlap	-2.4e-4/ -0.490	4.7e-5	46	-5.150	< 0.001 ***	-6.4e-3/ -0.556	2.8e-3	20	-2.334	0.030 *
Connectance → Niche Overlap	1.897/ 0.640	0.241	46	7.861	< 0.001 ***	-3.352/ -0.928	0.705	20	-4.757	< 0.001 ***
Nestedness → Niche Overlap	0.266/ 0.267	0.088	46	3.041	0.004 **	2.140/ 1.665	0.363	20	5.896	< 0.001 ***
Modularity → Niche Overlap	0.017/ 0.016	0.079	46	0.220	0.827	-0.443/ -0.058	1.070	20	-0.414	0.683
Global goodness-of-fit	Fisher's C = 6.543 , P = 0.038 on 2 Df					Fisher's C = 23.17 , P < 0.001 on 2 Df				

Supplementary Table 11. Stats of piecewise SEM on food webs whose dominant land-use type is farmland (n = 136 in terrestrial and n = 154 in aquatic system). The SEM dependency structure and the reported standardised estimates are corresponding to Supplementary Figure 1. Two-tailed significance code: · P < 0.1, * P < 0.05, ** P < 0.01, *** P < 0.001.

Land-use type specific model (analyse subset food webs with dominant land use as farmland)										
	Terrestrial					Aquatic				
**SEM dependencies**	Est./ Std.Est.	Std. Error	Df	t value	P value	Est./ Std.Est.	Std. Error	Df	t value	P value
Elevation → No.Nodes	0.058/ 0.296	0.016	134	3.590	< 0.001 ***	-5.8e-3/ -0.153	3.1e-3	152	-1.910	0.058 ·
Elevation → Connectance	-2.8e-6/ -0.108	1.7e-6	133	-1.616	0.108	-7.8e-5/ -0.442	1.3e-5	151	-5.960	< 0.001 ***
No.Nodes → Connectance	-8.5e-5/ -0.634	9.0e-6	133	-9.458	< 0.001 ***	-2.0e-4/ -0.044	3.4e-4	151	-0.591	0.555
Elevation → Nestedness	1.1e-5/ 0.169	5.9e-6	132	1.884	0.062 ·	-3.2e-5/ -0.095	1.3e-5	150	-2.432	0.016 *
No.Nodes → Nestedness	5.7e-5/ 0.170	3.9e-5	132	1.482	0.141	2.8e-3/ 0.323	3.1e-4	150	9.174	< 0.001 ***
Connectance → Nestedness	0.506/ 0.202	0.288	132	1.754	0.082 ·	1.492/ 0.787	0.073	150	20.37	< 0.001 ***
Elevation → Modularity	2.4e-5/ 0.228	7.8e-6	132	3.097	0.002 **	-1.8e-5/ -0.339	3.9e-6	150	-4.634	< 0.001 ***
No.Nodes → Modularity	3.7e-4/ 0.681	5.2e-5	132	7.211	< 0.001 ***	-8.0e-4/ -0.569	9.2e-5	150	-8.635	< 0.001 ***
Connectance → Modularity	1.507/ 0.369	0.385	132	3.910	< 0.001 ***	-0.057/ -0.189	0.022	150	-2.609	0.010 **
Elevation → Niche Overlap	-5.3e-5/ -0.550	5.0e-6	130	-10.65	< 0.001 ***	2.2e-5/ 0.108	9.0e-6	148	2.487	0.014 *
No.Nodes → Niche Overlap	-7.1e-5/ -0.143	3.7e-5	130	-1.898	0.060 ·	-1.2e-3/ -0.221	3.1e-4	148	-3.900	< 0.001 ***
Connectance → Niche Overlap	1.303/ 0.352	0.249	130	5.226	< 0.001 ***	-1.187/ -1.005	0.097	148	-12.28	< 0.001 ***
Nestedness → Niche Overlap	0.070/ 0.047	0.075	130	0.931	0.354	0.138/ 0.221	0.054	148	2.529	0.013 *
Modularity → Niche Overlap	-0.062/ -0.069	0.056	130	-1.117	0.266	-1.096/ -0.282	0.181	148	-6.047	< 0.001 ***
Global goodness-of-fit	Fisher’s C = 16.11, P < 0.001 on 2 Df					Fisher’s C = 12.99, P = 0.002 on 2 Df				

Supplementary Table 12. Stats of piecewise SEM on food webs whose dominant land-use type is urban area (n = 18 in terrestrial and n = 37 in aquatic system). The SEM dependency structure and the reported standardised estimates are corresponding to Supplementary Figure 1. Two-tailed significance code: · P < 0.1, * P < 0.05, ** P < 0.01, *** P < 0.001.

Land-use type specific model (analyse subset food webs with dominant land use as urban area)										
	Terrestrial					Aquatic				
**SEM dependencies**	Est./ Std.Est.	Std. Error	Df	t value	P value	Est./ Std.Est.	Std. Error	Df	t value	P value
Elevation → No.Nodes	-0.034/ -0.079	0.108	16	-0.318	0.754	7.9e-3/ 0.095	0.014	35	0.565	0.575
Elevation → Connectance	7.4e-6/ 0.164	9.5e-6	15	0.773	0.451	-1.3e-4/ -0.394	5.2e-5	34	-2.516	0.017 *
No.Nodes → Connectance	-5.5e-5/ -0.536	2.2e-5	15	-2.528	0.023 *	7.4e-4/ 0.186	6.3e-4	34	1.190	0.242
Elevation → Nestedness	8.3e-6/ 0.075	2.9e-5	14	0.283	0.781	1.4e-5/ 0.025	6.0e-5	33	0.239	0.812
No.Nodes → Nestedness	-3.2e-5/ -0.125	7.9e-5	14	-0.403	0.693	1.6e-3/ 0.235	6.7e-4	33	2.388	0.023 *
Connectance → Nestedness	0.344/ 0.139	0.781	14	0.440	0.666	1.324/ 0.775	0.180	33	7.349	< 0.001 ***
Elevation → Modularity	-8.9e-5/ -0.432	4.8e-5	14	-1.846	0.086 ·	-4.0e-5/ -0.291	2.0e-5	33	-1.991	0.055 ·
No.Nodes → Modularity	1.4e-4/ 0.258	1.3e-4	14	1.040	0.316	-7.1e-4/ -0.434	2.2e-4	33	-3.170	0.003 **
Connectance → Modularity	0.230/ 0.050	1.283	14	0.179	0.861	-0.157/ -0.383	0.060	33	-2.604	0.014 *
Elevation → Niche Overlap	-4.2e-5/ -0.284	3.6e-5	12	-1.147	0.274	-1.2e-5/ -0.035	2.6e-5	31	-0.438	0.664
No.Nodes → Niche Overlap	-2.2e-4/ -0.636	9.1e-5	12	-2.362	0.036 *	-7.5e-4/ -0.192	3.4e-4	31	-2.212	0.035 *
Connectance → Niche Overlap	0.017/ 0.005	0.856	12	0.020	0.984	-1.007/ -1.025	0.128	31	-7.899	< 0.001 ***
Nestedness → Niche Overlap	-0.058/ -0.044	0.305	12	-0.190	0.853	0.026/ 0.046	0.072	31	0.365	0.718
Modularity → Niche Overlap	-0.024/ -0.033	0.186	12	-0.127	0.901	-1.042/ -0.433	0.217	31	-4.804	< 0.001 ***
Global goodness-of-fit	Fisher’s C = 2.575, P = 0.276 on 2 Df					Fisher’s C = 0.462, P = 0.794 on 2 Df				

Supplementary Figures

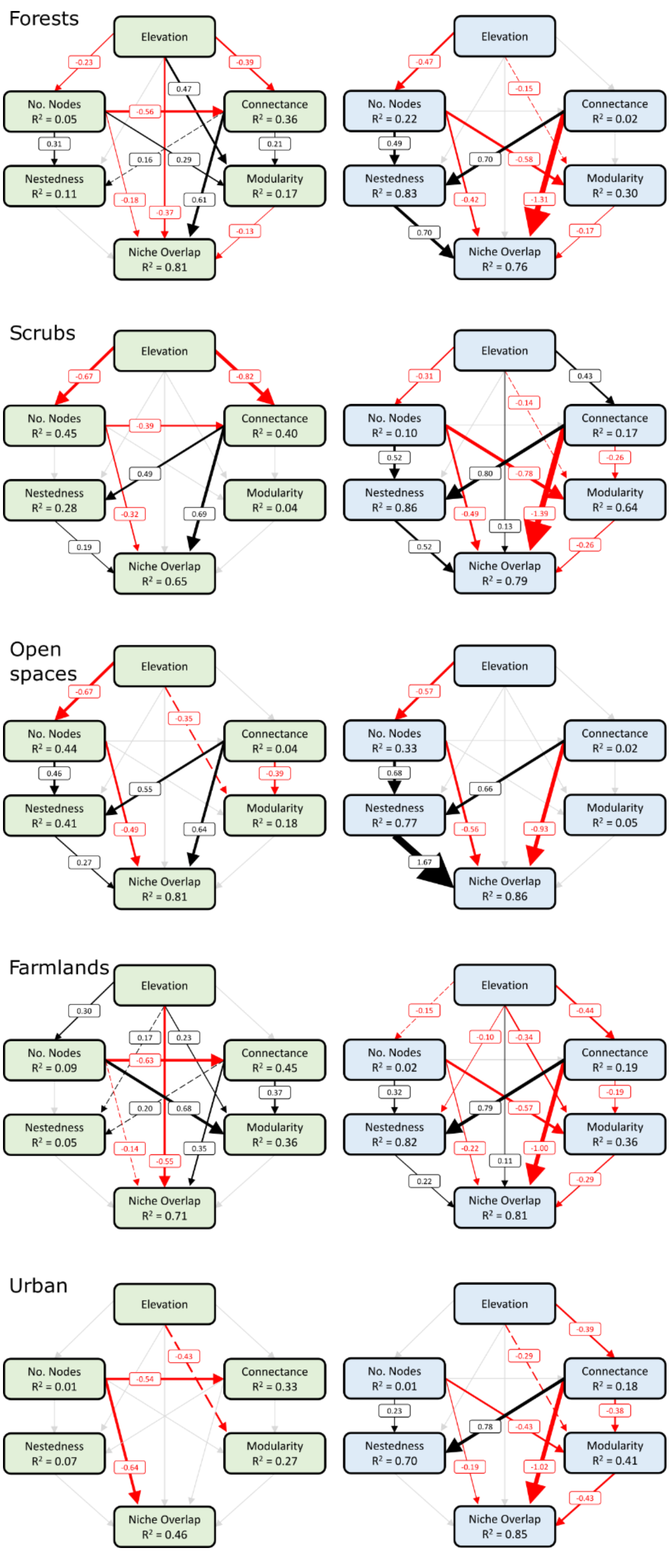

Supplementary Figure 1. Piecewise structural equation modelling (SEM) analyses as of the main text, but with subsetted food webs of each dominant land-use type. We note that most of the detected dependencies are subsets of the overall pattern (main text Figs. 3b–c), and farmlands exhibit significant yet opposite elevation to modularity and elevation to niche overlap influences in both green and blue food webs.

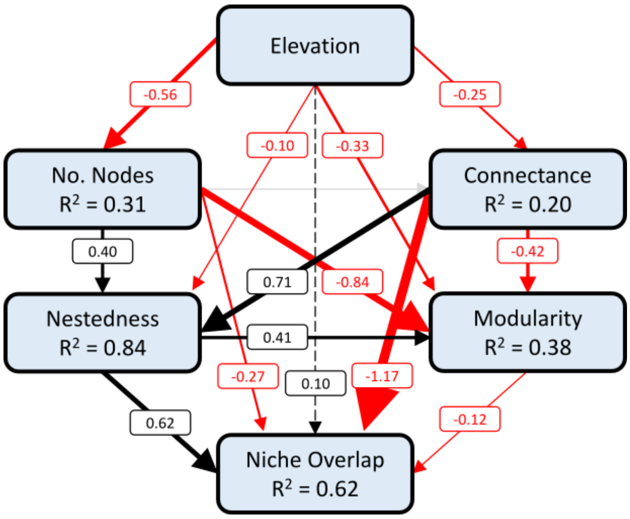

Supplementary Figure 2. Piecewise structural equation modelling (SEM) analyses of the blue food webs as of the main text, but with a direct path from nestedness to modularity, which is unspecified in our model structure (main text Fig. 3b). The analysis suggests that adding such a path better explains the data. We note that with or without this unspecified path, all other detected dependencies remain qualitatively and quantitatively consistent (main text Fig. 3c). Given such robustness, we present the results without this path in the main text, sticking with our literature-based model structure.

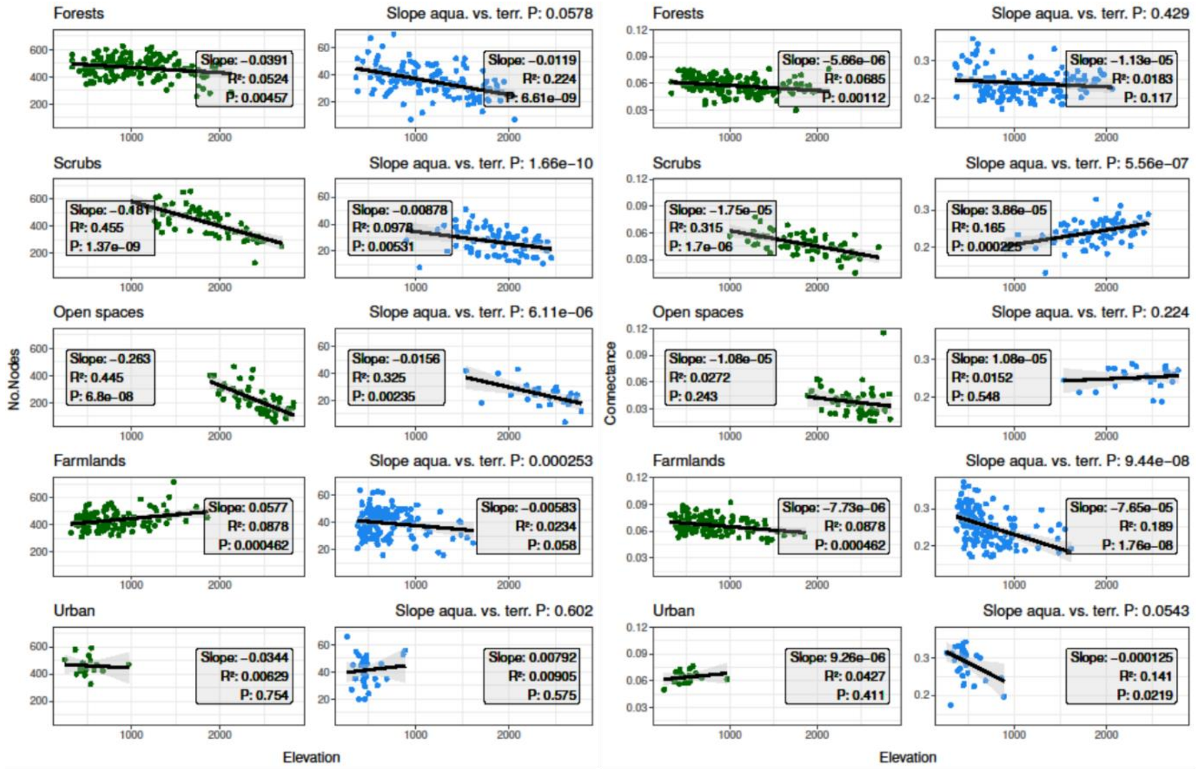

Supplementary Figure 3. Detailed slope comparisons with linear models testing the effects of elevation on number of nodes (left panel) and connectance (right panel) with subsetted inferred food webs of each dominant land-use type. Overlaying theses land-type specific plots gives the scatterplots in main text Fig. 4, while the two-tailed slope (and slope comparison) stats here are summarised in the barplots there. Solid lines and corresponding shades are the fitted regression and 95% CI, respectively.

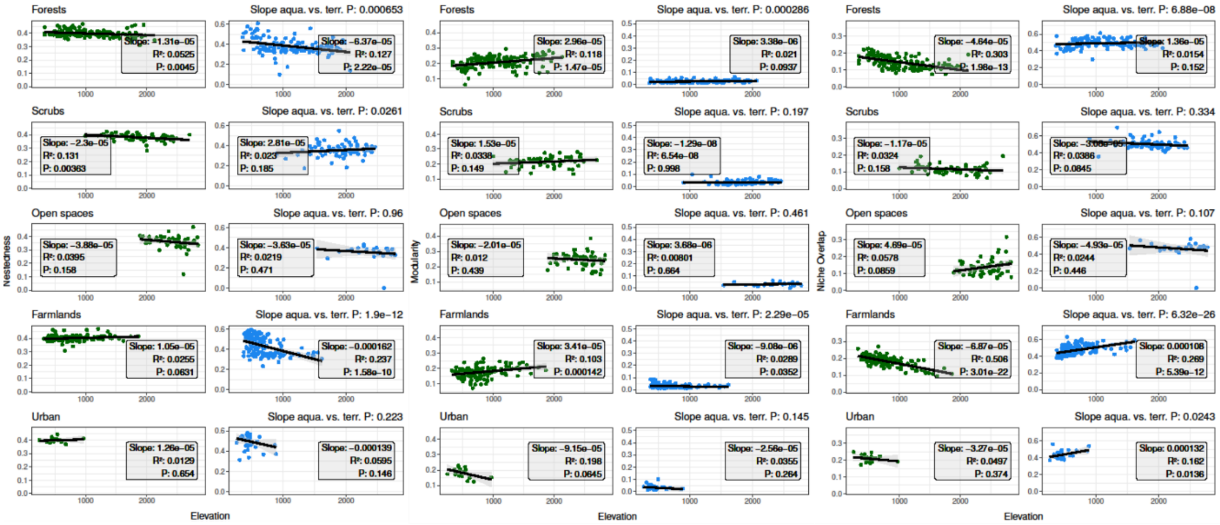

Supplementary Figure 4. Detailed slope comparisons with linear models testing the effects of elevation on nestedness (left panel), modularity (middle panel), and consumers' niche overlap (right panel) with subsetted inferred food webs of each dominant land-use type. Overlaying theses land-type specific plots gives the scatterplots in main text Fig. 5, while the two-tailed slope (and slope comparison) stats here are summarised in the barplots there. Solid lines and corresponding shades are the fitted regression and 95% CI, respectively.

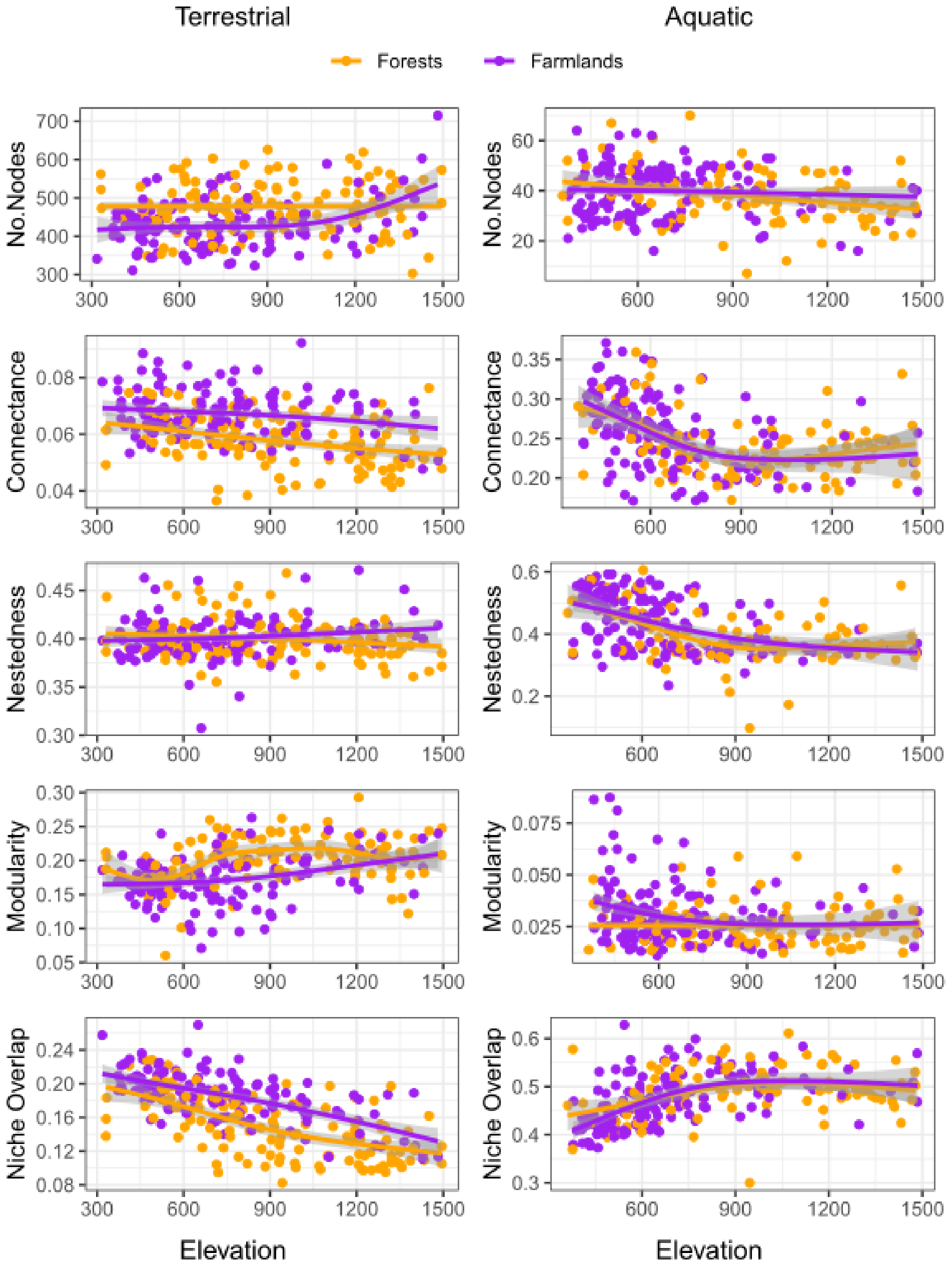

Supplementary Figure 5. Comparisons with generalised additive models testing the effects of elevation on all food-web properties among subsetted food webs in forests versus farmlands, underneath 1500 m elevation. These two land-use types overlap their distributions in such an elevational segment and are thus comparable within. Solid lines and corresponding shades are the fitted regression and 95% CI, respectively.

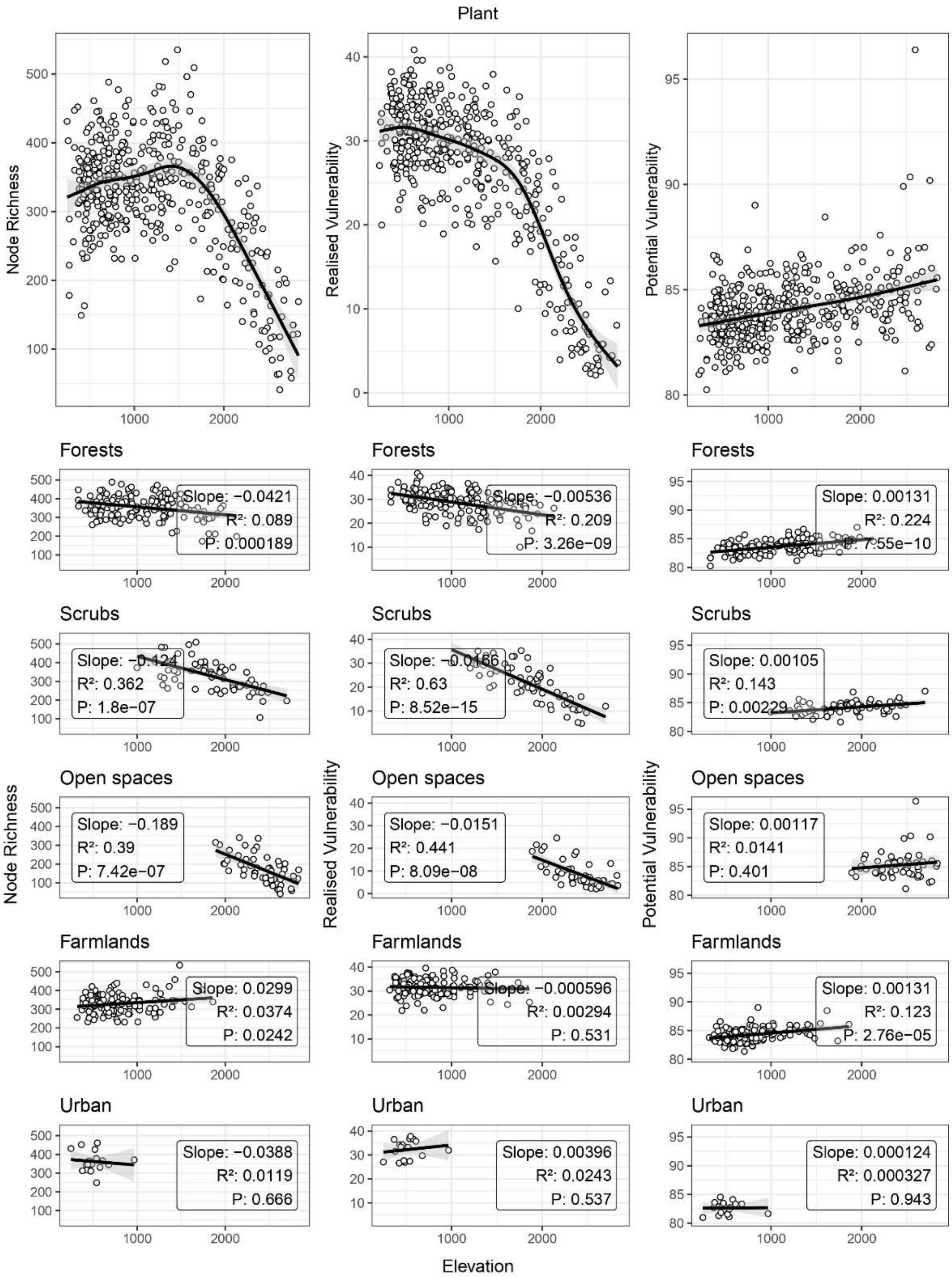

Supplementary Figure 6. Node richness (left panel), realised vulnerability (middle panel), and potential vulnerability (right panel) of plants in assembled green food webs along elevation. The realised vulnerability is how many consumers feed on each plant in an inferred food web (based on consumers' occurrence at each site), whereas the potential one is the same measure in the metaweb (regional integration of trophic interactions). Each dot represents the mean value of an inferred food web. The top plot shows the overall pattern, whereas the below are patterns partitioned based on each dominant land-use type. The black lines are the fitted regression of generalised additive (overall) or linear (land-type specific) models with the corresponding shades the 95% CI. Two-tailed regression slope stats are provided in the box.

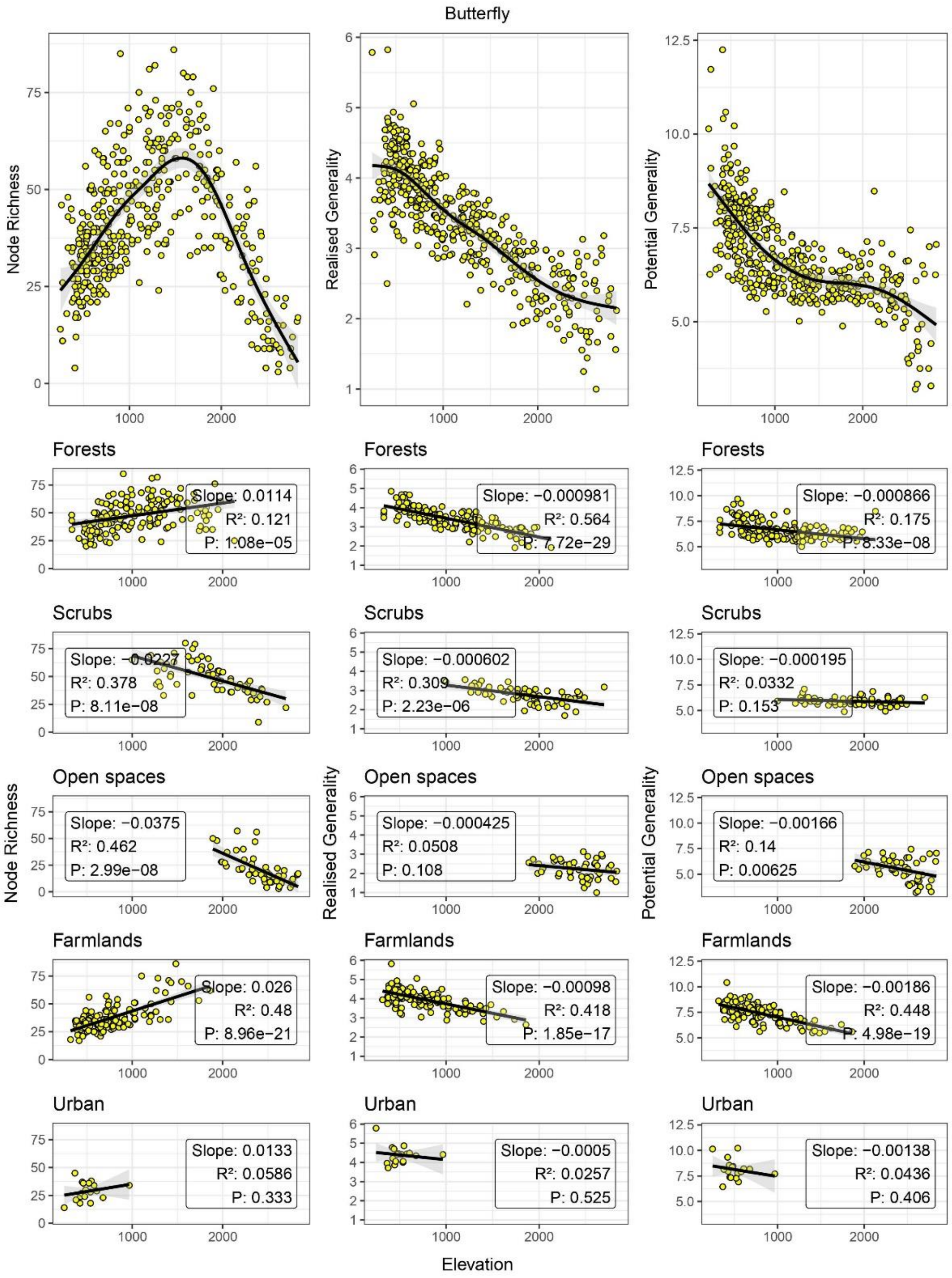

Supplementary Figure 7. Node richness (left panel), realised generality (middle panel), and potential generality (right panel) of butterfly larva in assembled green food webs along elevation. The realised generality is the number of resources (host plants) that each focal butterfly feeds on in an inferred food web (based on resources' occurrence at each site), whereas the potential one is the same measure in the metaweb (regional integration of trophic interactions, i.e., more its biological diet breadth). Each dot represents the mean value of an inferred food web. The top plot shows the overall pattern, whereas the below are patterns partitioned based on each dominant land-use type. The black lines are the fitted regression of generalised additive (overall) or linear (land-type specific) models with the corresponding shades the 95% CI. Two-tailed regression slope stats are provided in the box.

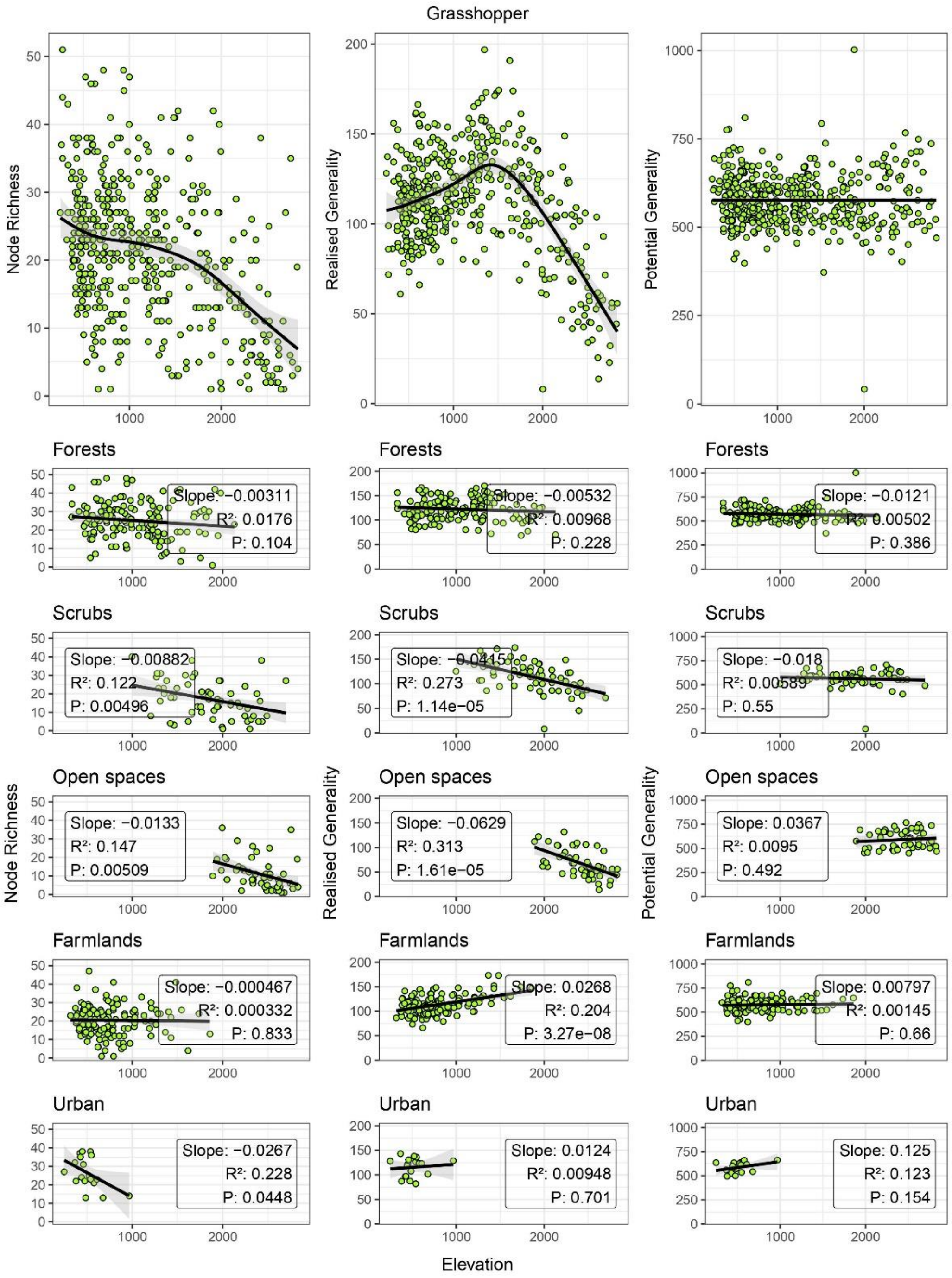

Supplementary Figure 8. Node richness (left panel), realised generality (middle panel), and potential generality (right panel) of grasshoppers in assembled green food webs along elevation. The realised generality is the number of resources that each focal grasshopper feeds on in an inferred food web (based on resources' occurrence at each site), whereas the potential one is the same measure in the metaweb (regional integration of trophic interactions, i.e., more its biological diet breadth). Each dot represents the mean value of an inferred food web. The top plot shows the overall pattern, whereas the below are patterns partitioned based on each dominant land-use type. The black lines are the fitted regression of generalised additive (overall) or linear (land-type specific) models with the corresponding shades the 95% CI. Two-tailed regression slope stats are provided in the box.

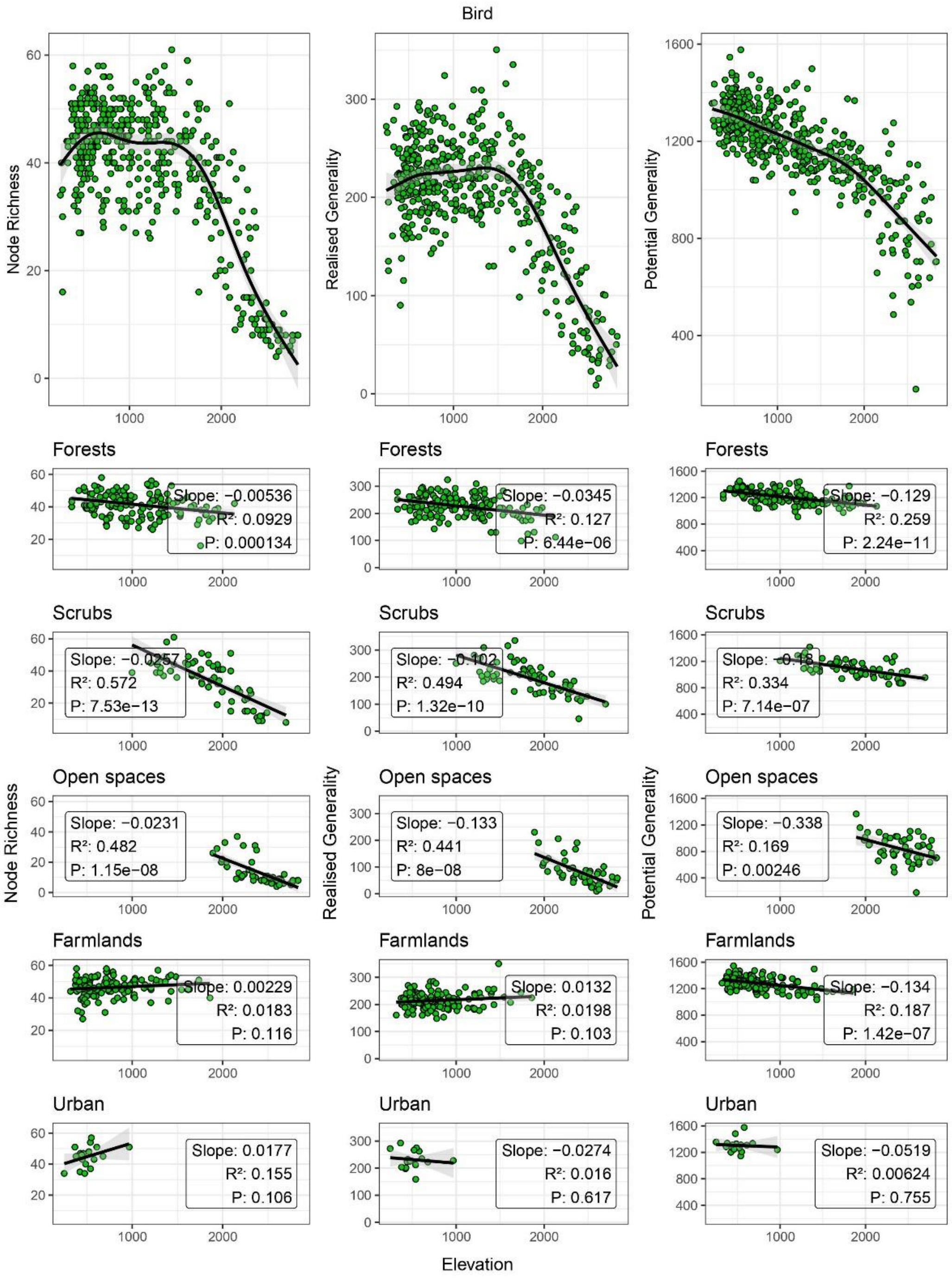

Supplementary Figure 9. Node richness (left panel), realised generality (middle panel), and potential generality (right panel) of birds in assembled green food webs along elevation. The realised generality is the number of resources that each focal bird feeds on in an inferred food web (based on resources' occurrence at each site), whereas the potential one is the same measure in the metaweb (regional integration of trophic interactions, i.e., more its biological diet breadth). Each dot represents the mean value of an inferred food web. The top plot shows the overall pattern, whereas the below are patterns partitioned based on each dominant land-use type. The black lines are the fitted regression of generalised additive (overall) or linear (land-type specific) models with the corresponding shades the 95% CI. Two-tailed regression slope stats are provided in the box.

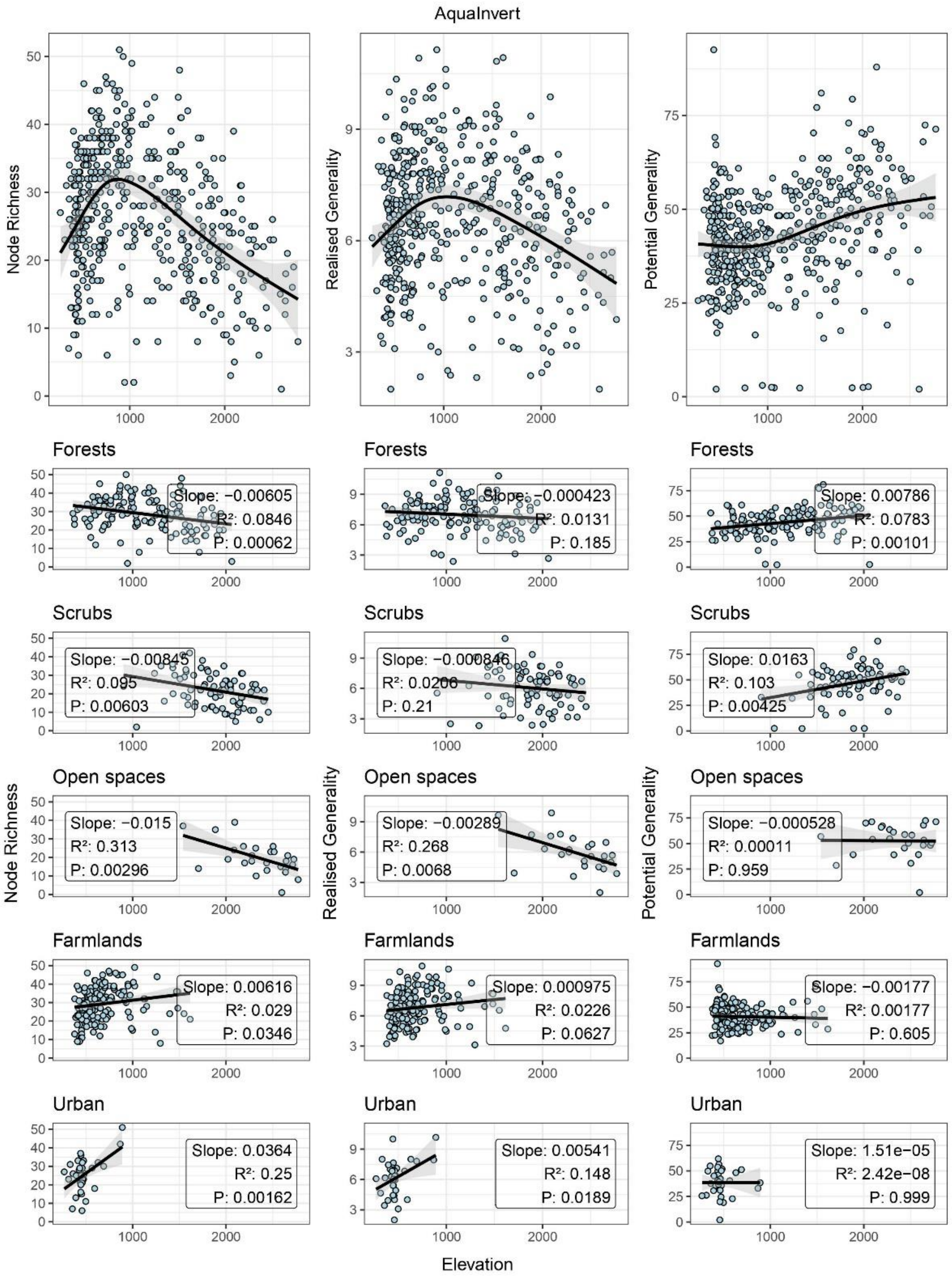

Supplementary Figure 10. Node richness (left panel), realised generality (middle panel), and potential generality (right panel) of aquatic invertebrates in assembled blue food webs along elevation. The realised generality is the number of resources that each focal invertebrate feeds on in an inferred food web (based on resources' occurrence at each site), whereas the potential one is the same measure in the metaweb (regional integration of trophic interactions, i.e., more its biological diet breadth). Each dot represents the mean value of an inferred food web. The top plot shows the overall pattern, whereas the below are patterns partitioned based on each dominant land-use type. The black lines are the fitted regression of generalised additive (overall) or linear (land-type specific) models with the corresponding shades the 95% CI. Two-tailed regression slope stats are provided in the box.

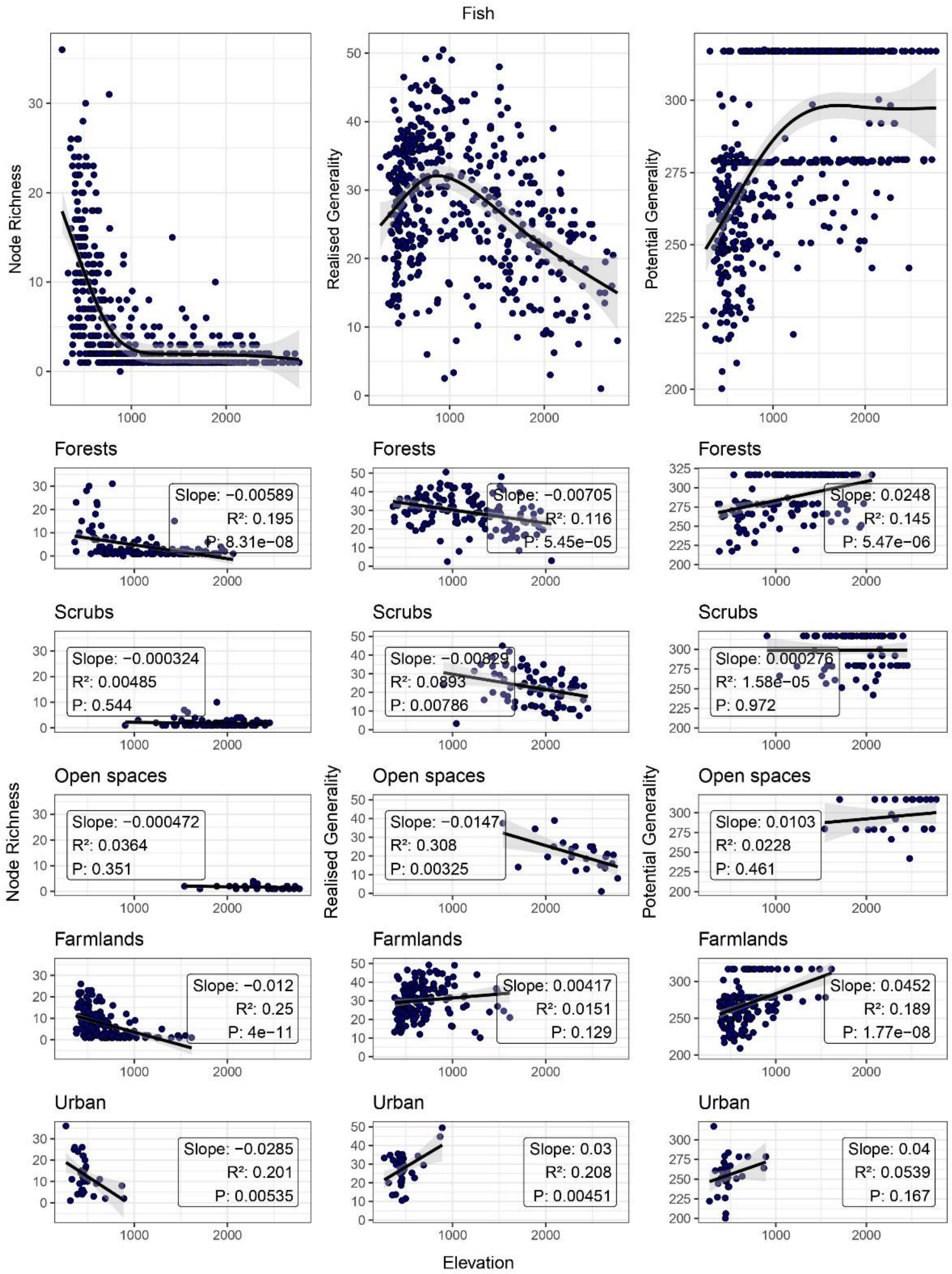

Supplementary Figure 11. Node richness (left panel), realised generality (middle panel), and potential generality (right panel) of fishes in assembled blue food webs along elevation. The realised generality is the number of resources that each focal fish consumes in an inferred food web (based on resources' local occurrence), whereas the potential one the same measure in the metaweb (more its biological diet breadth). Each dot represents the mean of an inferred food web. The top plot shows the overall pattern, whereas the below are partitioned by each dominant land-use type. The black lines are the fitted regression of generalised additive (overall) or linear (land-type specific) models with corresponding shades the 95% CI. Note that our 5×5 km^2 fish occurrence resolution and 1×1 km^2 grid-averaged elevation may assign more fish species than actual occurring to some sites, particularly high-elevation ones. Two-tailed regression slope stats are provided in the box.

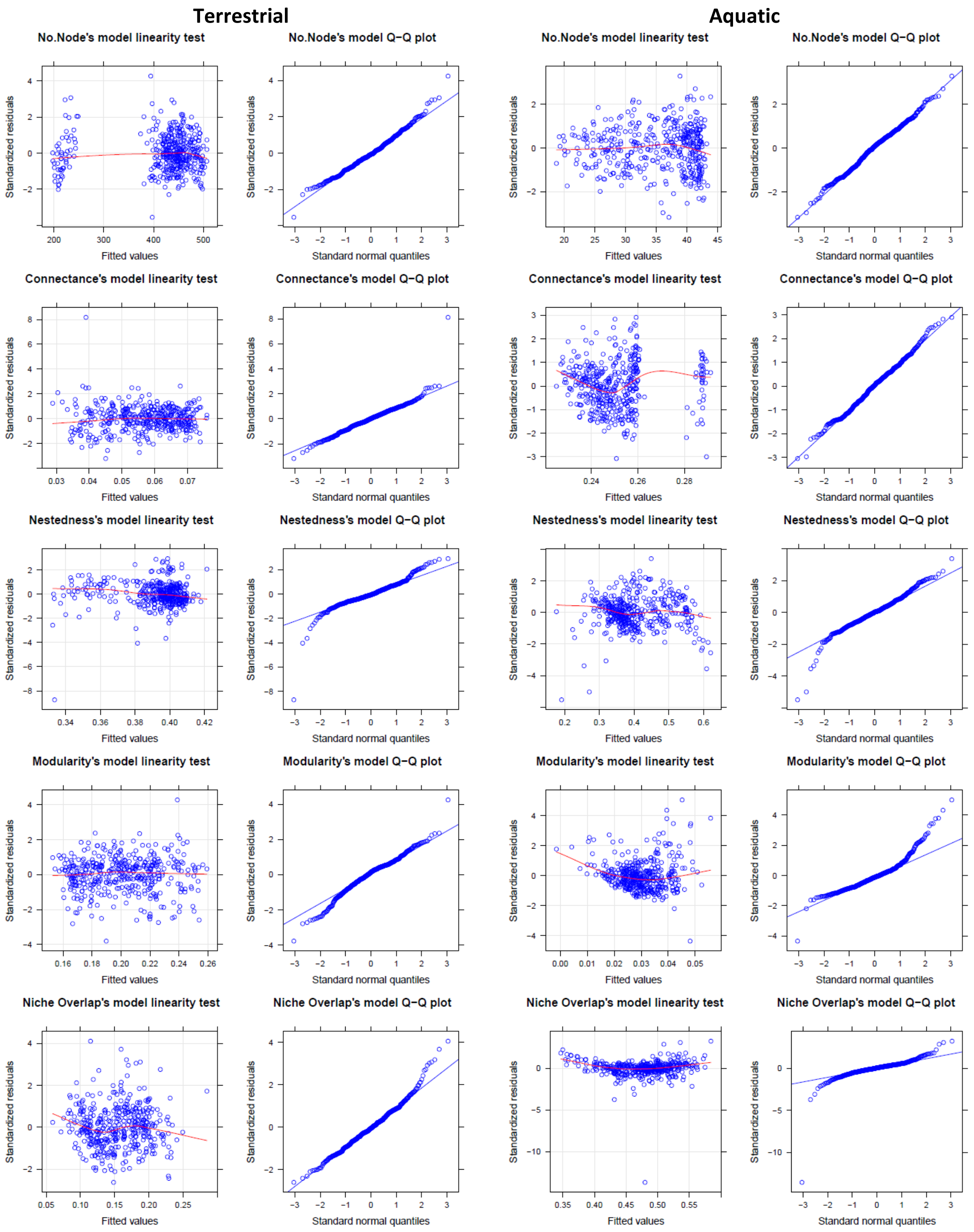

Supplementary Figure 12. Assumption tests of the linear mixed models (LMM) embedded in the piecewise SEM analyses. This is of the overall SEM for all inferred food webs with definable dominant land-use type (n = 421 in terrestrial and n = 430 in aquatic system). While some assumptions were violated in individual cases, in the majority of cases they were met. Based on the principle of parsimony, we thus considered using LMM-embedded SEM a valid method. Same for the land-use type specific analyses (see Supplementary Figures 13–17).

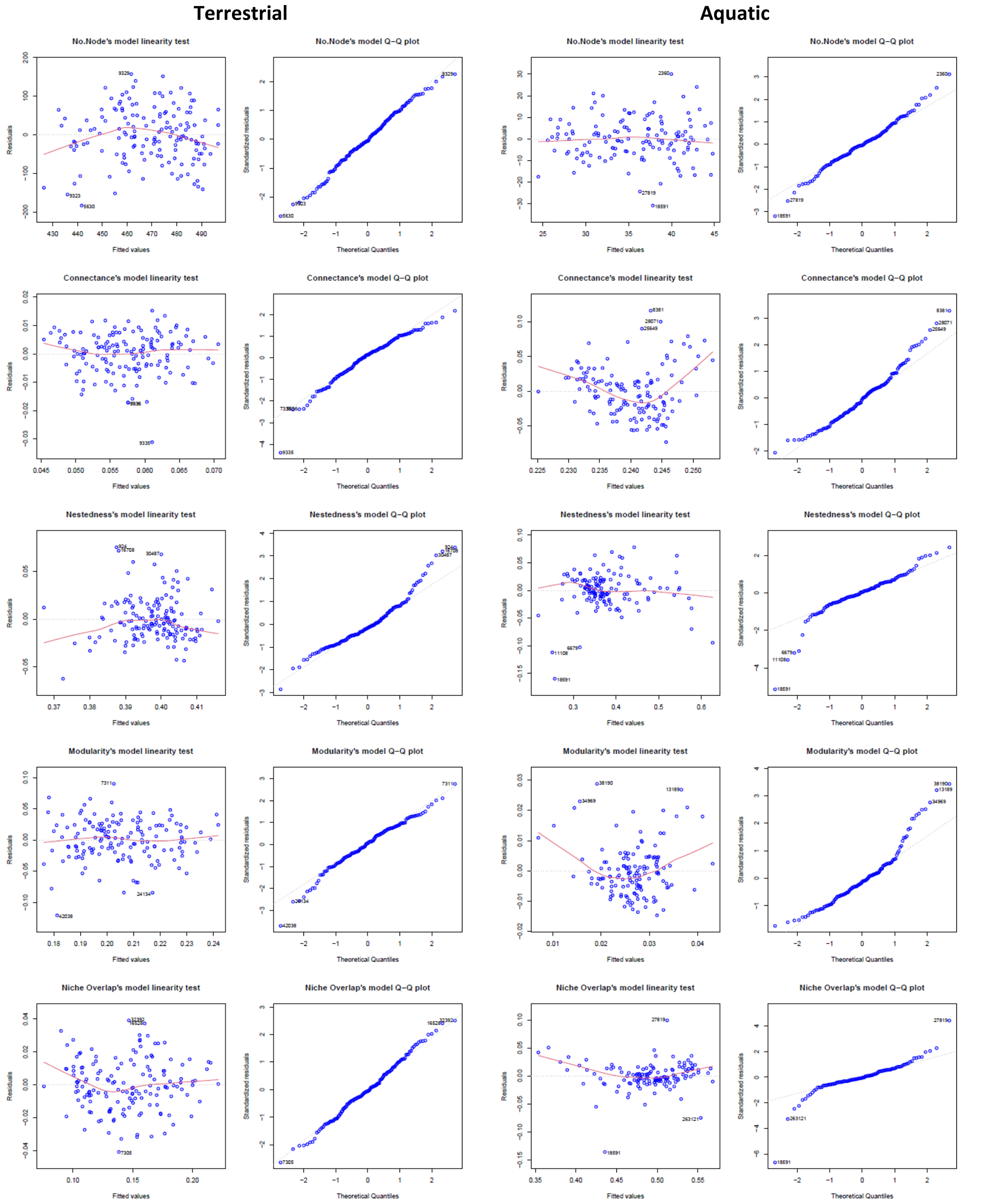

Supplementary Figure 13. Assumption tests of the linear mixed models embedded in the piecewise SEM analyses. This is of the land-use specific SEM for inferred food webs whose dominant land-use type is forest (n = 152 in terrestrial and n = 135 in aquatic system). See the caption of Supplementary Figure 12 for more information.

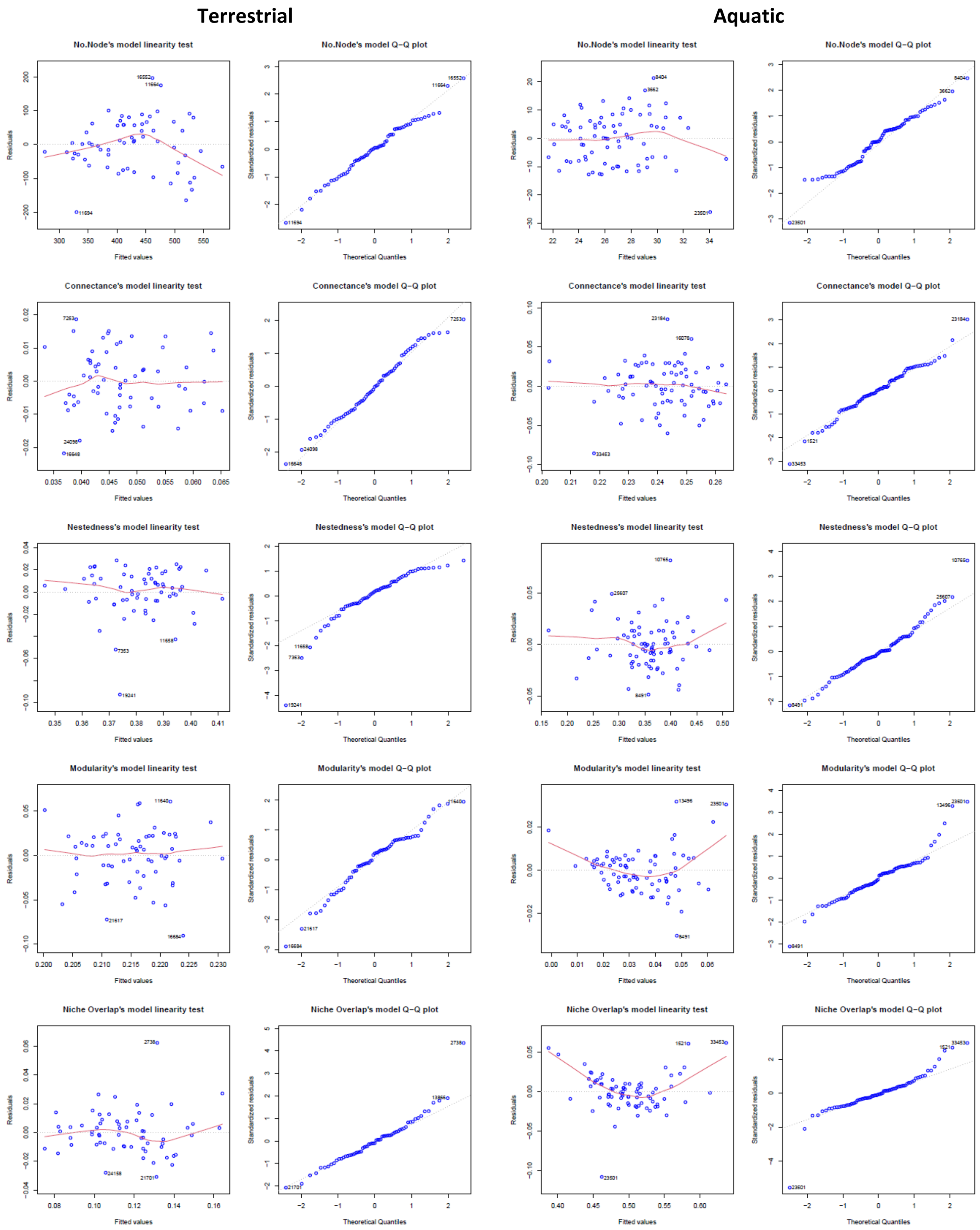

Supplementary Figure 14. Assumption tests of the linear mixed models embedded in the piecewise SEM analyses. This is of the land-use specific SEM for inferred food webs whose dominant land-use type is scrub (n = 63 in terrestrial and n = 78 in aquatic system). See the caption of Supplementary Figure 12 for more information.

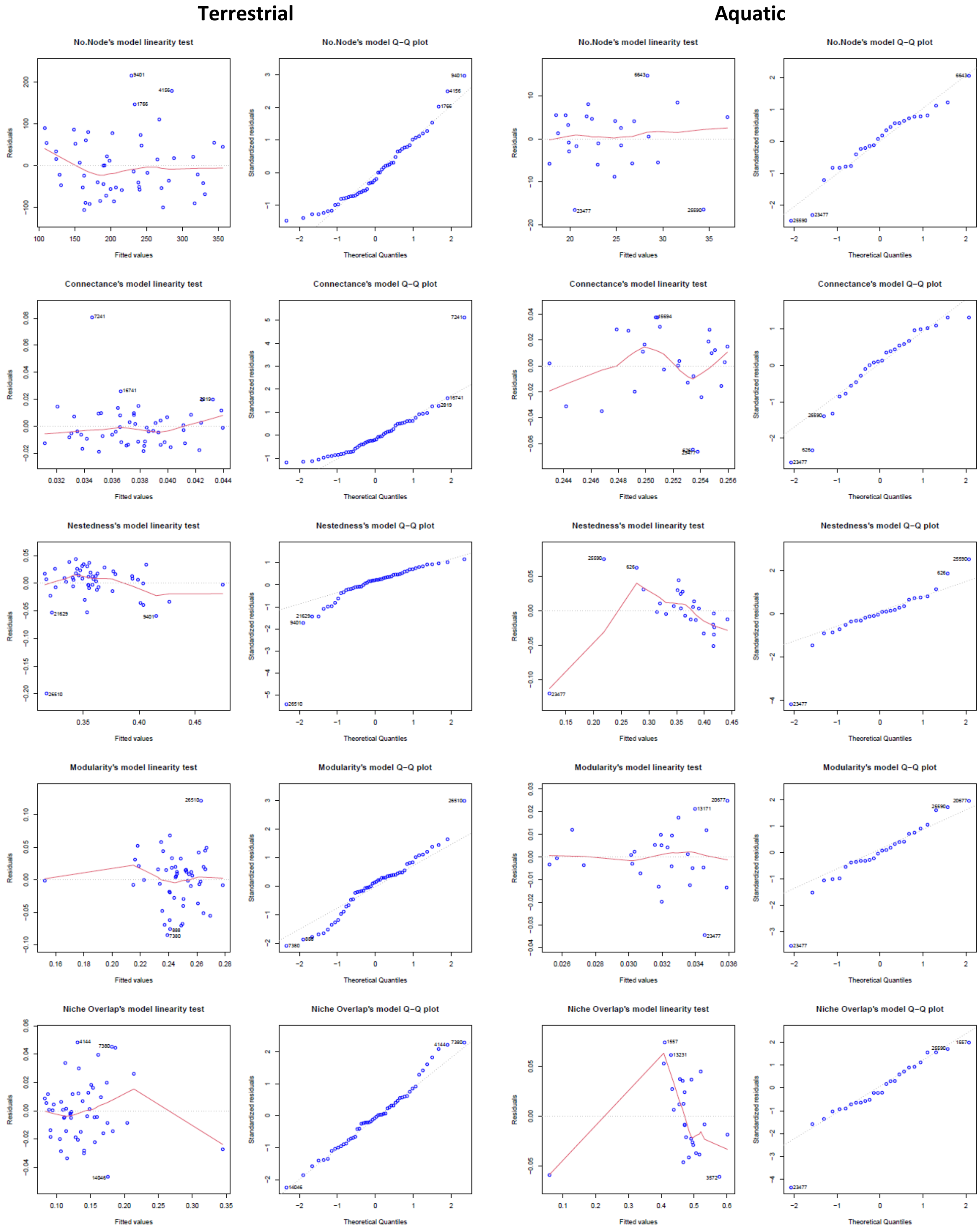

Supplementary Figure 15. Assumption tests of the linear mixed models embedded in the piecewise SEM analyses. This is of the land-use specific SEM for inferred food webs whose dominant land-use type is open space (n = 52 in terrestrial and n = 26 in aquatic system). See the caption of Supplementary Figure 12 for more information.

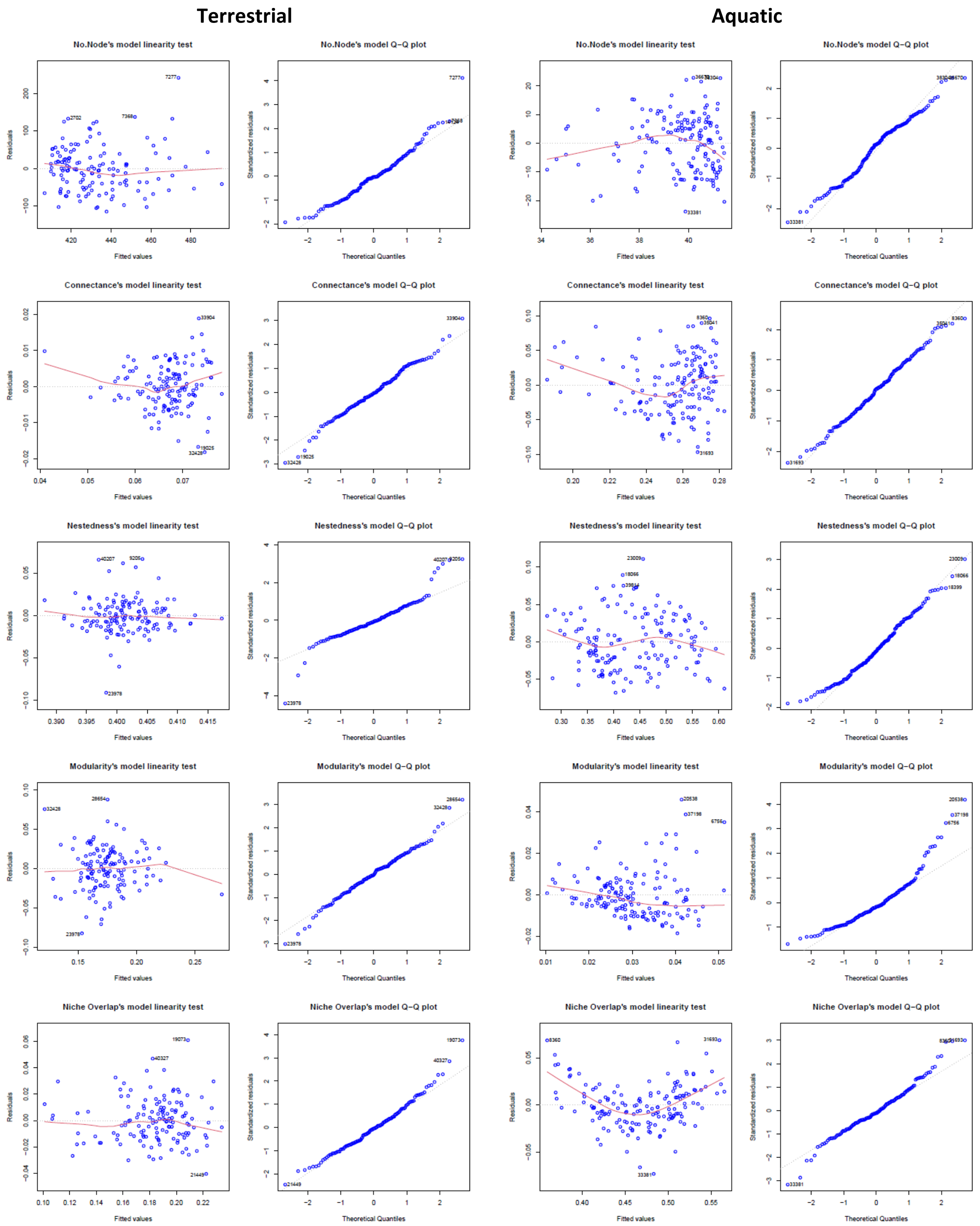

Supplementary Figure 16. Assumption tests of the linear mixed models embedded in the piecewise SEM analyses. This is of the land-use specific SEM for inferred food webs whose dominant land-use type is farmland (n = 136 in terrestrial and n = 154 in aquatic system). See the caption of Supplementary Figure 12 for more information.

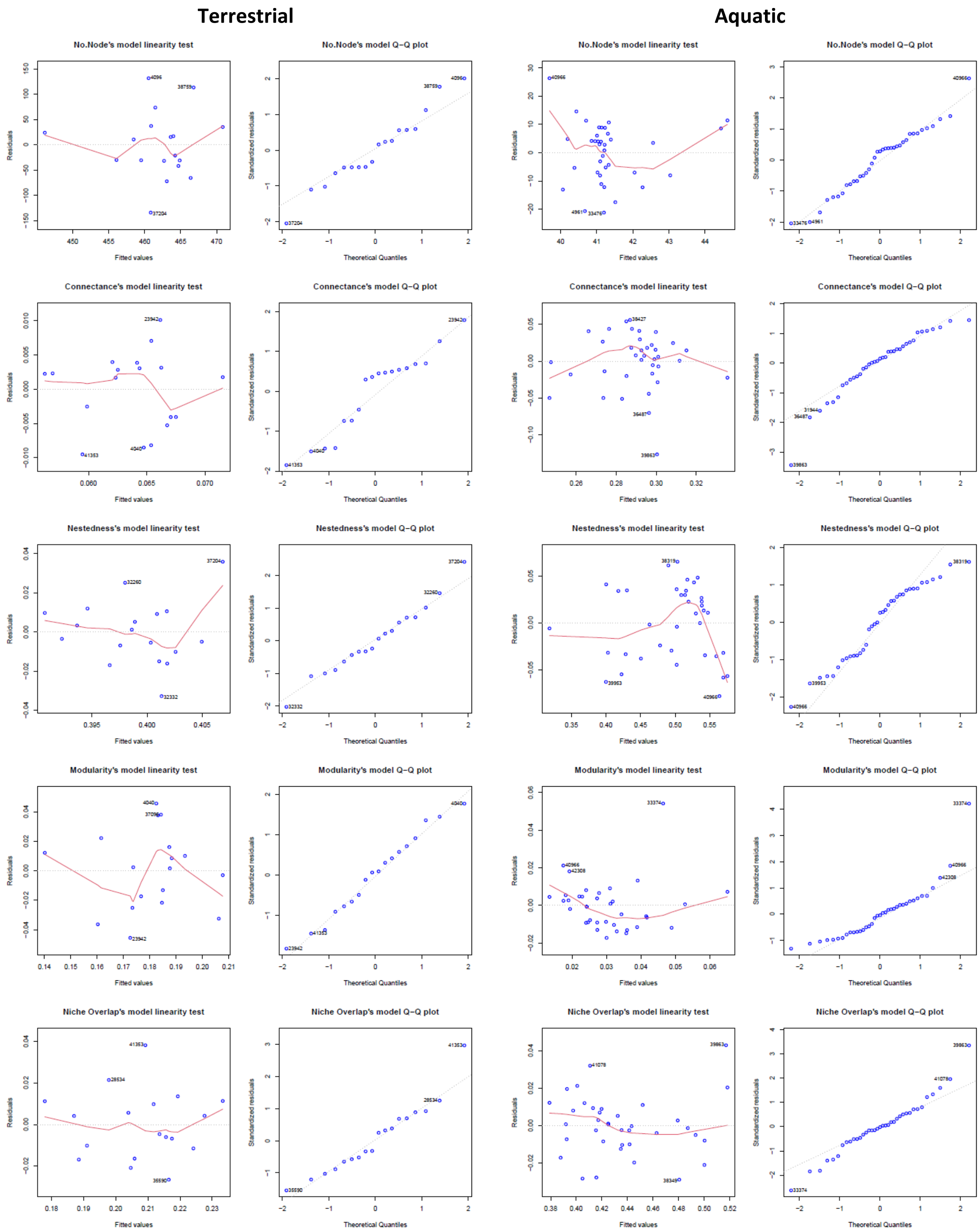

Supplementary Figure 17. Assumption tests of the linear mixed models embedded in the piecewise SEM analyses. This is of the land-use specific SEM for inferred food webs whose dominant land-use type is urban area (n = 18 in terrestrial and n = 37 in aquatic system). See the caption of Supplementary Figure 12 for more information.

Supplementary Discussion

Blue-green merged food webs

In addition to the blue and green food webs analysed in the main text, we also constructed “blue-green merged” food webs. The sympatric (i.e., co-occurring at the same grid) blue and green taxa were connected based on our trophic understandings of birds’ preying on fishes and/or aquatic invertebrates (provided in Supplementary Methods). Although the blue and green taxa occurrences were each representatively monitored at randomly selected sites, such site selection needed to fulfil respective constraints of the two systems (e.g., the blue survey must be conducted at where water flows), so the blue and green sites were not designed to be aligned. Moreover, data from different sources were not be spatially aligned neither. Our complied dataset finds 94 grids where all blue and green taxa groups occur and some defined blue-green trophic interactions happen at a rougher 5×5 km^2 grid resolution, while none at a 1×1 km^2 resolution. We therefore conducted the blue-green merged food-web analyses at the 5×5 km^2 resolution (i.e., occurrences aggregated and environmental measurements averaged over 25 neighbouring 1×1 km^2 grids). We note that, however, with this lower resolution, the representativeness of taxa occurrence and environmental measurements become much lower, and the number of grids where we could define dominant land-use type (see main text *Methods*) also become quite few (1 urban, 31 farmlands, 17 forests, 6 scrubs, and 5 open spaces). Therefore, we here focus mainly on the spatial structural analyses with corresponding GAMs (see main text *Methods*). Also, our trophic understandings (i.e., metaweb) did not cover other potentially existing blue-green interactions (e.g., between blue and green invertebrates). We thus do not claim the associations between environmental and biotic components are as firm as the ones in the main text.

In brief, the results showed that the merged food webs exhibited qualitatively similar structural changes along elevation as in green food webs (Supplementary Figure 18, with reference to main text Fig. 4 & 5). This is not surprising, given that in our dataset the green food webs had considerably more number of nodes than the blue ones (main text Fig. 4; arguably biologically realistic, but also partly contributed by our lower taxonomic resolution and usage of mega-node basal resources in the blue webs). When connecting local blue and green webs together, the green nodes and links would be the predominant components and thus the main structure shaper of the merged product. This, however, does not imply that the blue taxa play relatively non-important roles in a blue-green interconnected ecosystem. On the one hand, given the significance of blue-green boundary, we believe that the structure of blue-green merged food webs would be better understood via an interconnected multi-layered perspective (e.g., Kivelä et al. 2014). This way, potentially, the respective essence of the two systems, e.g., the very different number of taxa, would be contained within each of their own layer, and the overall structure would not be biased toward the system with more nodes. However, we have yet seen solid structure quantifying measurement be developed in the field of multi-layered networks, thus we here could only merge the blue and green food webs as a single-layered one. On the other hand, we believe that it is the overall population dynamics where the cross-system trophic interactions matter the most. Species that connect

the two systems may actually play important ecological roles that associate the population dynamics of both, thereby determining species coexistence or ecological functions of the merged system. While relevant meta-ecosystem theoretical models exist (Osakpolor et al., 2021), to empirically investigate the dynamics of a merged system, one would need to monitor time-series population abundance fluctuations therein, as well as the corresponding temporal food-web structural change. This is unfortunately beyond the scope of what our data could address. To our knowledge, empirical exploration of the trophic interactions (*per se*) across blue-green boundary is already difficult and thus remains scarce, not to mention tracking them within a food-web context. Nonetheless, there are novel methods emerging, e.g., analysing fatty acid composition (Kowarik et al., 2021), to allow cross-boundary monitoring of trophic interactions. We hope progress as such would soon allow relevant data to be collected, revealing the detailed dynamics of blue-green connected food webs at least at a localised scale.

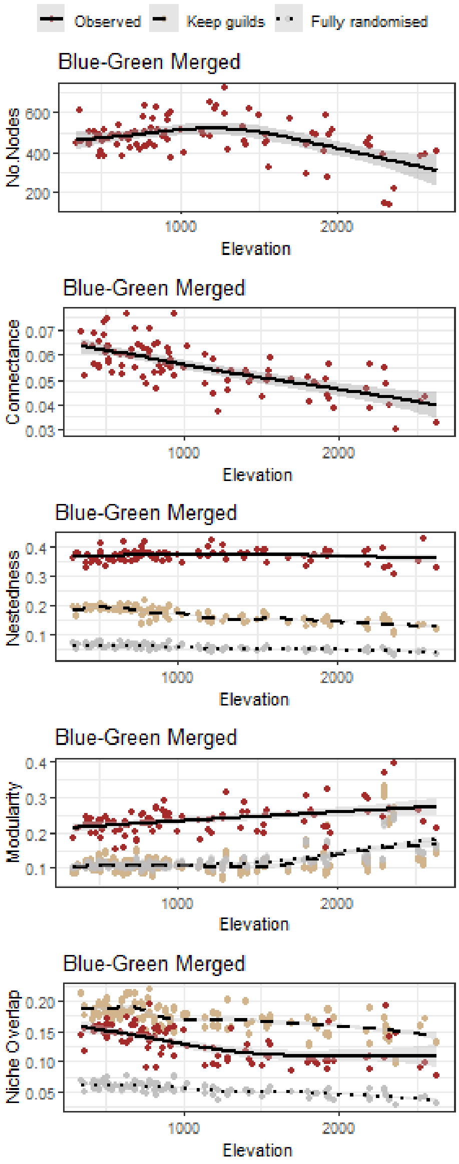

Supplementary Figure 18. Number of nodes, connectance, nestedness, modularity, and consumers' niche overlap of blue-green merged food webs along elevation. As in main text Fig. 4 & 5, the black lines (solid, dashed, and dotted) are the fitted regression of generalized additive models with corresponding shades the 95% CI. For definition of "keep guilds" and "fully randomised" schemes, see main text *Methods*.

Sensitivity analyses

With the metaweb approach, our constructed local food webs were inferences based on trophic interactions from literature, with the assumption that these interactions will realise if the interacting taxa co-occur. This assumption draws a realistic boundary of potential interactions within which we know realisable interactions exist, leading to a non-biased comparison of potential food webs across local sites. Meanwhile, this necessarily ignores possible intraspecific diet variation as detailed in main text *Methods*. It is possible that some trophic interactions recorded and reported in the literature will not realise at a given local site due to certain localised constraints. In other words, the local food webs we constructed may contained links that do not realise.

To check if this potential overestimation of trophic-link realisation by the metaweb approach would change our findings, we performed a series of sensitivity analyses to mirror the main analyses of the study. For each inferred (i.e., observed) local food web, we randomly removed 10% of its links to mimic those being overestimated by the metaweb. Any node that became isolated due to the link removal was subsequently removed, too. This was repeated five times per web as replicates. We then performed the same principal component analysis (PCA), piecewise structural equation modelling (SEM), and generalised additive model (GAM) analyses on these generated webs as we did with the original observed webs in the main text.

As for the results, all of the PCA, piecewise SEM, and GAM analyses (Supplementary Figure 19) showed qualitatively and quantitatively very similar outcomes to those of our main text analyses on the inferred food webs (main text Fig. 3—5). The way that blue and green food web structurally different from each other (i.e., green webs have more nodes, are more nodular and less connected) was in line with main text results (Supplementary Figure 19a). In the piecewise SEM, the majority of the identified significant dependencies among elevation and food-web properties were qualitatively (in terms of the sign of coefficients), and even somewhat quantitatively (in terms of the size of coefficients), the same as the main text results (Supplementary Figure 19b). There were two dependencies in the blue food webs that were negative in the main text analyses here became positive (i.e., elevation to nestedness, and modularity to niche overlap, Supplementary Figure 19b). These were nonetheless rather weak dependencies and their changing signs were likely driven by the changing size of coefficients pointing toward the respective response food-web properties, especially given that the sensitivity analyses here have five-time larger sample size than the main analyses so would be more sensitive to detecting weak effects. In the GAM analyses, all five focal food-web metrics respond to the elevation the same way as in our main text analyses (Supplementary Figure 19c). Here again, as the sample size became five-time larger, the land-use-specific linear-model slope comparisons detected more significant slopes than in the main text analyses, but they are qualitatively in line with each other (Supplementary Figure 19c bottom panel).

In summary, all the main findings that we pointed out and discussed in the main text remained qualitatively consistent with the sensitivity analyses. This indicates that our findings were robust to a potential overestimation of link realisation of the metaweb approach.

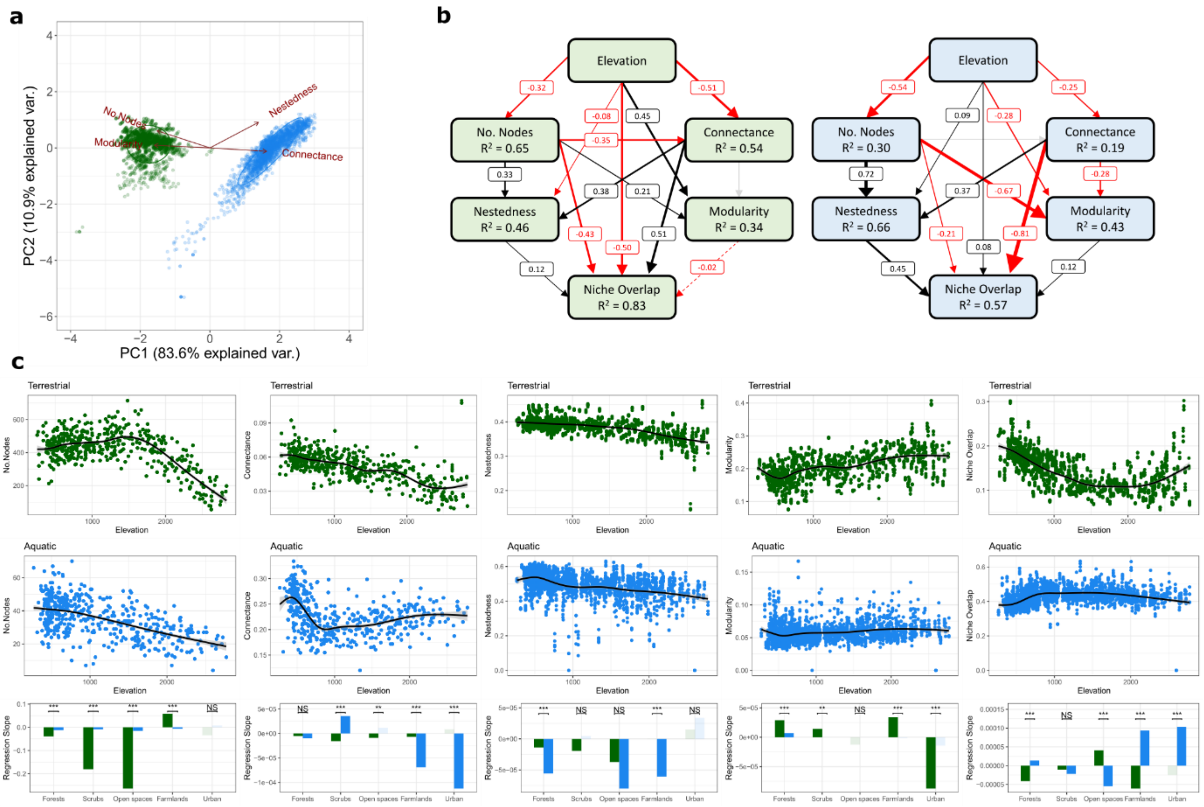

Supplementary Figure 19. Sensitivity analyses results with food webs whose 10% links were randomly removed (in comparison to the observed webs) to mimic the potential local inaccuracy of a metaweb. The results of **(a)** PCA analysis, **(b)** piecewise SEM analyses, and **(c)** GAM analyses with land-use-specific linear-model slope comparisons were mostly qualitatively consistent with the results presented in main text Fig. 3—5. All figure annotations (e.g., error bands, significant codes) are consistent with main text Fig. 3—5, please refer to the captions there for respective definition.

Supplementary References

1. Aphalo, Pedro J. "ggpmisc: Miscellaneous Extensions to 'ggplot2'". R package version 0.3.7. (2020).
2. AQEM expert consortium, 2002. Ecological classifications by AQEM expert consortium, www.aqem.de
3. Auguie, Baptiste "gridExtra: Miscellaneous Functions for "Grid" Graphics". R package version 2.3. (2017).
4. Bates D, Mächler M, Bolker B, Walker S. "Fitting Linear Mixed-Effects Models Using lme4." Journal of Statistical Software. (2015): 67(1), 1–48.
5. Baur, Bertrand, and Bern Naturhistorisches Museum. "Die Heuschrecken der Schweiz". Haupt, (2006).
6. Cantor, Mauricio, et al. "Nestedness across biological scales." PloS one 12.2 (2017): e0171691.
7. Csardi, Gabor, and Tamas Nepusz. "The igraph software package for complex network research." InterJournal, complex systems 1695.5 (2006): 1-9.
8. Detzel, Peter. "Die Heuschrecken Baden-Württembergs". 51 Tabellen. Ulmer, (1998).
9. Dormann, Carsten F., et al. "Indices, graphs and null models: analyzing bipartite ecological networks." The Open Ecology Journal 2.1 (2009).
10. Ebert, Günter. "Die Schmetterlinge Baden-Württembergs" Vol. 1–10. Eugen Ulmer, Stuttgart (1991–2005).
11. Froese, Rainer, and Daniel Pauly. "FishBase." (2010).
12. Graf, Wolfram, et al. "Distribution and ecological preferences of European freshwater organisms". Volume 1. Trichoptera. Vol. 1. Schmidt-Kloiber A, Hering D (Series editors). Pensoft Publishing, (2008).
13. Graf, Wolfram, et al. "Distribution and Ecological Preferences of European Freshwater Organisms". Volume 2. Plecoptera. Vol. 2. Schmidt-Kloiber A, Hering D (Series editors). Pensoft Publishing, (2009).
14. Ingrisch, Sigfrid, and Günter Köhler. "Die Heuschrecken Mitteleuropas." (1998): 94.
15. Kassambara, Alboukadel. "ggpubr: 'ggplot2' Based Publication Ready Plots." R package version 0.4.0. (2020).
16. Kivelä, M., Arenas, A., Barthelemy, M., Gleeson, J. P., Moreno, Y., & Porter, M. A. "Multilayer networks". Journal of Complex Networks (2014), 2(3), 203-271.
17. Klaiber, J., Altermatt, F., Birrer, S., Chittaro, Y., Dziock, F., Gonseth, Y., Hoess, R., Keller, D., Kuechler, H., Luka, H., Manzke, U., Mueller, A., Pfeifer, M.A., Roesti, C., Schlegel, J., Schneider, K., Sonderegger, P., Walter, T., Holderegger, R., Bergamini, A. (2017). "Fauna Indicativa". WSL Berichte, 54. ISSN: 2296-3456
18. Kottelat, Maurice, and Jorg Freyhof. Handbook of European freshwater fishes. Publications Kottelat, (2007).
19. Kowarik, C., Martin-Creuzburg, D., & Robinson, C. T. "Cross-ecosystem linkages: Transfer of polyunsaturated fatty acids from streams to riparian spiders via emergent insects". Frontiers in Ecology and Evolution, 438 (2021).
20. Landolt, Elias, et al. "Flora indicativa: Okologische Zeigerwerte und biologische Kennzeichen zur Flora der Schweiz und der Alpen". Haupt (2010).
21. Lefcheck, Jonathan S. "piecewiseSEM: Piecewise structural equation modelling in r for ecology, evolution, and systematics." Methods in Ecology and Evolution 7.5 (2016): 573-579.
22. Lenth, Russell V. "Least-squares means: the R package lsmeans." Journal of statistical software 69.1 (2016): 1-33.

23. Maas, Stephan, Peter Detzel, and Aloysius Staudt. "Gefährdungsanalyse der Heuschrecken Deutschlands: Verbreitungsatlas, Gefährdungseinstufung und Schutzkonzepte". Ergebnisse aus dem F+ E-Vorhaben 89886015 des Bundesamtes für Naturschutz. na, (2002).
24. Moog, Otto. "Fauna aquatica austriaca." Wasserwirtschaftskataster, Bundesministerium für Land-und Forstwirtschaft, Wien (1995).
25. Osakpolor, S. E., Kattwinkel, M., Schirmel, J., Feckler, A., Manfrin, A., & Schäfer, R. B. "Mini-review of process-based food web models and their application in aquatic-terrestrial meta-ecosystems". Ecological Modelling 458 (2021), 109710.
26. Pitteloud, Camille, et al. "The structure of plant–herbivore interaction networks varies along elevational gradients in the European Alps." Journal of Biogeography 48.2 (2021): 465-476.
27. Sarkar D. Lattice: Multivariate Data Visualization with R. Springer, New York (2008).
28. Schlumprecht, Helmut, and Georg Waeber. "Heuschrecken in Bayern." Verlag Eugen Ulmer, Stuttgart (2003).
29. Schmedtje, U., and M. Colling. "Ökologische Typisierung der aquatischen Makrofauna Bayrisches Landesamt für Wasserwirtschaft." München, Germany (1996).
30. Schmidt-Kloiber, Astrid, and Daniel Hering. "www.freshwaterecology.info–An online tool that unifies, standardises and codifies more than 20,000 European freshwater organisms and their ecological preferences." Ecological indicators 53 (2015): 271-282.
31. Storchová, Lenka, and David Hořák. "Life-history characteristics of European birds." Global Ecology and Biogeography 27.4 (2018): 400-406.
32. Tachet, Henri, et al. "Invertébrés d'eau douce: systématique, biologie, écologie". Vol. 15. Paris: CNRS éditions, (2010).
33. Urbanek, Simon. “png: Read and write PNG images.” R package version 0.1-7. (2013).
34. Vu, Vincent Q. "ggbiplot: A ggplot2 based biplot." R package version 0.55 755. (2011).
35. Wickham, Hadley. “ggplot2: Elegant Graphics for Data Analysis.” Springer-Verlag New York, (2016).
36. Wood, S. “Generalized additve models: an introduction with R”. Chapman and Hall/CRC, New York, New York, USA (2006).
37. Wood, S. “Fast stable restricted maximum likelihood and marginal likelihood estimation of semiparametric generalized linear models”. Journal of the Royal Statistical Society (B) (2011): 73(1):3-36
38. Yu, Guangchuang. “scatterpie: Scatter Pie Plot.” R package version 0.1.6. (2021).
39. Zuur, A. F., E. N. Ieno, N. Walker, A. A. Saveliev, and G. M. Smith. “Mixed effects models and extensions in ecology with R”. Springer, New York, New York, USA (2009).
