# Supplementary material for: Blue and green food webs respond differently to elevation and land use
Source: Nat Commun. 2022 Oct 27;13:6415. doi: 10.1038/s41467-022-34132-9 (PMC9613893; doi:10.1038/s41467-022-34132-9)
Supplement: Supplementary file 3 — Reporting Summary [file 41467_2022_34132_MOESM3_ESM.pdf]

nature portfolio

Corresponding author(s): Hsi-Cheng Ho
Florian Altermatt

Last updated by author(s): Sep 21, 2022

Reporting Summary

Nature Portfolio wishes to improve the reproducibility of the work that we publish. This form provides structure for consistency and transparency in reporting. For further information on Nature Portfolio policies, see our Editorial Policies and the Editorial Policy Checklist.

Statistics

For all statistical analyses, confirm that the following items are present in the figure legend, table legend, main text, or Methods section.

n/a	Confirmed	
☐	☒	The exact sample size (n) for each experimental group/condition, given as a discrete number and unit of measurement
☐	☒	A statement on whether measurements were taken from distinct samples or whether the same sample was measured repeatedly
☐	☒	The statistical test(s) used AND whether they are one- or two-sided<br>*Only common tests should be described solely by name; describe more complex techniques in the Methods section.*
☒	☐	A description of all covariates tested
☐	☒	A description of any assumptions or corrections, such as tests of normality and adjustment for multiple comparisons
☐	☒	A full description of the statistical parameters including central tendency (e.g. means) or other basic estimates (e.g. regression coefficient) AND variation (e.g. standard deviation) or associated estimates of uncertainty (e.g. confidence intervals)
☐	☒	For null hypothesis testing, the test statistic (e.g. F, t, r) with confidence intervals, effect sizes, degrees of freedom and P value noted<br>*Give P values as exact values whenever suitable.*
☒	☐	For Bayesian analysis, information on the choice of priors and Markov chain Monte Carlo settings
☒	☐	For hierarchical and complex designs, identification of the appropriate level for tests and full reporting of outcomes
☒	☐	Estimates of effect sizes (e.g. Cohen's d, Pearson's r), indicating how they were calculated

Our web collection on statistics for biologists contains articles on many of the points above.

Software and code

Policy information about availability of computer code

Data collection	For data collection, no software was used.
Data analysis	All data analyses were performed under R version 4.0.3 (R Core Team). All applied packages and functions were described in Supplementary Information Section S2. These include: UNODF package (ver. 1.0), the graph.adjacency, multilevel.community, and modularity functions of igraph package (ver. 1.3.4), the networklevel function of bipartite package (ver. 2.17), R-base prcomp function, the ggbiplot function of ggbiplot package (ver. 0.55), R-base lm and anova functions, the psem function of piecewiseSEM package (ver. 2.1.2), the lmer function of lme4 package (ver. 1.1-30), the xyplot and qqmath functions of lattice package (ver. 0.20-45), R-base plot function, the ggplot2 package (ver. 3.3.6), the lstrends function of lsmeans package (ver. 2.30-0), the gam function of mgcv package (ver. 1.8.40), the geom_scatterpie function from scatterpie package (ver. 0.1.7), the png function from png package (ver. 0.1-7), the ggpubr (ver. 0.4.0), ggpmisc (ver. 0.4.7), and gridExtra (ver. 2.3) packages. The R codes performing analyses can be accessed at this public depository: https://figshare.com/s/e3bd3d8c0b8078252986.

For manuscripts utilizing custom algorithms or software that are central to the research but not yet described in published literature, software must be made available to editors and reviewers. We strongly encourage code deposition in a community repository (e.g. GitHub). See the Nature Portfolio guidelines for submitting code & software for further information.

Data

Policy information about availability of data

All manuscripts must include a data availability statement. This statement should provide the following information, where applicable:

- Accession codes, unique identifiers, or web links for publicly available datasets
- A description of any restrictions on data availability
- For clinical datasets or third party data, please ensure that the statement adheres to our policy

Taxa occurrence (from databases/datasets of BDM: https://www.biodiversitymonitoring.ch/, info fauna: http://www.cscf.ch/cscf/, and Progetto Fiumi Project of Eawag) and GIS environmental information (from databases including DHM25: https://www.swisstopo.admin.ch/en/geodata/height/dhm25.html, CLC: https://land.copernicus.eu/pan-european/corine-land-cover, and CHELSA: http://chelsa-climate.org) are data that we obtained from respective authorities in charge (as listed in the Methods sectioni), which shall be accessed via contacting these authorities. Source Data are provided with this paper. These include the processed metaweb trophic-interaction data and local food-web data, which are also accessible at this public depository: https://figshare.com/s/e3bd3d8c0b8078252986.

Field-specific reporting

Please select the one below that is the best fit for your research. If you are not sure, read the appropriate sections before making your selection.

☐ Life sciences ☐ Behavioural & social sciences ☒ Ecological, evolutionary & environmental sciences

For a reference copy of the document with all sections, see nature.com/documents/nr-reporting-summary-flat.pdf

Ecological, evolutionary & environmental sciences study design

All studies must disclose on these points even when the disclosure is negative.

Study description	Based on knowledge-based trophic interaction metaweb and empirically recorded taxa occurrence (focal taxonomic groups: bird, grasshopper, butterly, plant, fish, and aquatic invertebrate), we inferrentially constructed local terretrial and aquatic (stream) food webs across the area of Switzerland. We analysed and revealed the patterns of various food-web properties (structural and ecological ones) along elevation gradient and among different key land-use types.
Research sample	Occurrence data of the focal taxonomic groups (birds, grasshoppers, butterflies, plants, fishes and aquatic invertebrates) were provided by respective monitoring authorities (as listed above) on a 1x1 km grid basis (details see Methods). These represent the spatial (co-)occurrence of relevant species where they could form ecological communities. Trophic interaction information was compiled by the authors based on literature and expert knowledge, as provided in the Supplementary Information Section S1.
Sampling strategy	N/A (our study is an analysis of existing/published data, so no actual sampling was conducted. The sample sizes, i.e., number of local food webs, were determined by the overlapping data grids across the taxa occurrence datasets.)
Data collection	For taxa occurrence, envrionmental factors, and trophic interactions, we based our information compilation and analyses on existing/published data or literature, so did not perform actual data collection (i.e., observations recording) ourselves. Details of data collection shall be obtained from the information sources (e.g., literature or database authorities) cited in the manuscript. For taxa occurrence, to the authors' understanding, the relevant authorieties performed standardised empirical surveys conducted by trained biologists with fixed protocols. For estabilishing the metaweb, Hsi-Cheng Ho, Florian Altermatt, Silvana Kaeser and Merin Reji Chacko compiled trophic-interaction knowledge (noting who eats whom as the metaweb provided in the Source Data) from various literature.
Timing and spatial scale	The timing of occurrence data have variation among taxa, but in general spans the period of Jan. 2001- Dec, 2020. The GIS environmental information is averaged over the period of Jan. 2005- Dec. 2015. Together, they reflect a long-term yet modern (i.e., not from the last century) understanding of the biotic components and abiotic condition of local communities. The focal spatial scale is the whole area of Switzerland.
Data exclusions	No data excluded
Reproducibility	This study includes no experiments. With the Source Data and codes provided, all results can be reproduced.
Randomization	The sampling sites are based on the Swiss Biodiversity Monitoring, 462 and 465 sites of terrestrial and aquatic sites sampled, respectively. The sampling sites are positioned based on a stratified random site selection (details see Swiss Biodiversity Monitoring).
Blinding	N/A (Our study is an analysis of existing/published long-term monitoring data, where bliding is irrelevant.)

Did the study involve field work? ☐ Yes ☒ No

Reporting for specific materials, systems and methods

We require information from authors about some types of materials, experimental systems and methods used in many studies. Here, indicate whether each material, system or method listed is relevant to your study. If you are not sure if a list item applies to your research, read the appropriate section before selecting a response.

Materials & experimental systems

n/a	Involved in the study
☒	☐ Antibodies
☒	☐ Eukaryotic cell lines
☒	☐ Palaeontology and archaeology
☒	☐ Animals and other organisms
☒	☐ Human research participants
☒	☐ Clinical data
☒	☐ Dual use research of concern

Methods

n/a	Involved in the study
☒	☐ ChIP-seq
☒	☐ Flow cytometry
☒	☐ MRI-based neuroimaging
